# Functional roles of LaeA, polyketide synthase, and glucose oxidase in the regulation of ochratoxin A biosynthesis and virulence in *Aspergillus carbonarius*

**DOI:** 10.1111/mpp.13013

**Published:** 2020-11-10

**Authors:** Uriel Maor, Omer Barda, Sudharsan Sadhasivam, Yang Bi, Elena Levin, Varda Zakin, Dov B. Prusky, Edward Sionov

**Affiliations:** ^1^ Institute of Postharvest and Food Sciences The Volcani Center Agricultural Research Organization Rishon LeZion Israel; ^2^ Institute of Biochemistry, Food Science and Nutrition The Robert H. Smith Faculty of Agriculture, Food and Environment The Hebrew University of Jerusalem Rehovot Israel; ^3^ College of Food Science and Engineering Gansu Agricultural University Lanzhou China

**Keywords:** *Aspergillus carbonarius*, GOX, LaeA, OTA biosynthesis, PKS, postharvest disease

## Abstract

*Aspergillus carbonarius* is the major producer of ochratoxin A (OTA) among *Aspergillus* species, but the contribution of this secondary metabolite to fungal virulence has not been assessed. We characterized the functions and addressed the roles of three factors in the regulation of OTA synthesis and pathogenicity in *A. carbonarius*: LaeA, a transcriptional factor regulating the production of secondary metabolites; polyketide synthase, required for OTA biosynthesis; and glucose oxidase (GOX), regulating gluconic acid (GLA) accumulation and acidification of the host tissue during fungal growth. Deletion of *laeA* in *A. carbonarius* resulted in significantly reduced OTA production in colonized nectarines and grapes. The ∆*laeA* mutant was unable to efficiently acidify the colonized tissue, as a direct result of diminished GLA production, leading to attenuated virulence in infected fruit compared to the wild type (WT). The designed *Acpks*‐knockout mutant resulted in complete inhibition of OTA production in vitro and in colonized fruit. Interestingly, physiological analysis revealed that the colonization pattern of the ∆*Acpks* mutant was similar to that of the WT strain, with high production of GLA in the colonized tissue, suggesting that OTA accumulation does not contribute to *A. carbonarius* pathogenicity. Disruption of the *Acgox* gene inactivated GLA production in *A. carbonarius*, and this mutant showed attenuated virulence in infected fruit compared to the WT strain. These data identify the global regulator LaeA and GOX as critical factors modulating *A. carbonarius* pathogenicity by controlling transcription of genes important for fungal secondary metabolism and infection.

## Graphical abstract

LaeA and GOX are critical factors modulating *A. carbonarius* pathogenicity in fruits by controlling transcription of genes important for fungal secondary metabolism and infection; OTA does not contribute to *A. carbonarius* virulence.

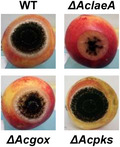

## 1 INTRODUCTION

*Aspergillus* species are widespread fungal pathogens in nature, exhibiting a saprophytic lifestyle in the soil and on a wide range of substrates, including foods and feeds. Several species are among the typical pathogens of harvested fruit and vegetables (Barkai‐Golan, 2001). Most *Aspergillus* species occur in foods as spoilage or biodeterioration fungi (Hocking, 2007). Black *Aspergillus* species appear to be secondary invaders, following other fungal pathogens and insects that produce mechanical damage on fruit tissues (Varga and Kozakiewicz, 2006). *Aspergillus carbonarius* is frequently responsible for the postharvest decay of various fresh fruit, including grapes, peaches, pears, citrus, and nectarines, as well as grains, coffee, and nuts. Windborne spores from the soil are deposited onto the surface of those fruit (Battilani et al., 2006). The fungus frequently penetrates the commodities through harvesting wounds and bruises, or damage caused by preharvest rain, mechanical impact, and insects. *A. carbonarius* is associated with maturing fruit and is responsible for a large portion of the economic losses sustained during storage and shipment. In addition to its saprophytic colonization, the pathogen is also the main producer of ochratoxin A (OTA) in grapes, dried vine fruit, and wine, especially in Mediterranean countries (Covarelli et al., 2012; Gil‐Serna et al., 2018a). OTA is a potent nephrotoxin with carcinogenic, teratogenic, and immunotoxic properties in animals and possibly humans. There are several major parameters governing fungal growth and OTA production; some of them are intrinsic factors such as water availability, pH, and the composition of the substrate, and others are extrinsic factors such as temperature, moisture, and the competitive endogenous microbiota (Bellí et al., 2004; Lasram et al., 2010; Mitchell et al., 2004). Not much is known about the specific factors contributing to *A. carbonarius* colonization in fruit. Maor et al. (2017) demonstrated that ambient pH plays an important role in *A. carbonarius* pathogenicity and OTA biosynthesis. Secretion of gluconic acid (GLA) by *A. carbonarius* caused direct acidification of fruit tissue and induced OTA accumulation in colonized grapes (Maor et al., 2017). Previous findings have indicated that acidification of the apple fruit host environment through secretion of organic acids enhances the activation of pathogenicity factors, maceration, and colonization of the fruit by *Penicillium expansum* (Barad et al., 2012; Prusky et al., 2004). However, it is not clear if the similar acidification process induced during *A. carbonarius* infection contributes only to OTA production, the colonization process by the pathogen, or both.

Many studies have shown the involvement of global transcription factors in the regulation of secondary metabolite biosynthesis in filamentous fungi, most predominantly LaeA (Bok and Keller, 2004). This transcription factor was first identified in *Aspergillus nidulans*, but is now known to be conserved in all filamentous ascomycetes (Jain and Keller, 2013). LaeA has been found to be a positive regulator of most common mycotoxins, including sterigmatocystin (Bok and Keller, 2004), aflatoxin (Kale et al., 2008), fumonisin (Butchko et al., 2012), cyclopiazonic acid (Georgianna et al., 2010), trichothecenes (Kim et al., 2013), citrinin, patulin (Kumar et al., 2018; Liu et al., 2016), and OTA (Crespo‐Sempere et al., 2013). In *A. carbonarius*, much work has been carried out to understand the biosynthetic pathway and accumulation of OTA (Gallo et al., 2009, 2012, 2014). However, its relation and contribution to pathogen colonization are less clear. To our knowledge, mechanisms of pathogenicity and OTA biosynthesis during fruit colonization by *A. carbonarius* have not been addressed. Here, we characterized the importance of the transcription factor LaeA, a polyketide synthase (PKS) of the OTA‐biosynthesis pathway, and glucose oxidase (GOX), which have recently been identified in the *A. carbonarius* genome, in regulating synthesis of the mycotoxin OTA, and their contribution to colonization of nectarines and grapes by *A. carbonarius*. For the first time, we demonstrate that pathogenicity in *A. carbonarius* is regulated by LaeA and GOX, which mediate the differential expression of genes encoding cell wall‐degrading enzymes that cause host tissue maceration.

## 2 RESULTS

### 2.1 Disruption of *AclaeA*, *Acpks*, and *Acgox* genes

To explore the functional roles of LaeA (accession no. OOF95411), PKS (accession no. OOF93599), and GOX (accession no. OOF95573) in the physiology and pathogenicity of *A. carbonarius*, deletion strains *AclaeA*, *Acpks*, and *Acgox* were generated. A targeted gene‐deletion strategy was employed using *Agrobacterium tumefaciens*‐mediated transformation of *A. carbonarius* wild‐type (WT) strain NRRL 368 (Figures S1a, S2a, and S3a). Gene‐replacement plasmids pRFHU2‐AclaeA, pRFHU2‐Acpks, and pRFHU2‐Acgox were obtained by the USER‐friendly cloning system. Cocultivation of *A. tumefaciens* cells carrying the desired plasmid with the conidia of *A. carbonarius* led to the appearance of hygromycin B‐resistant colonies approximately 4 days after transfer to selective potato dextrose agar (PDA) plates. Disruption of *AclaeA*, *Acpks*, and *Acgox* genes was confirmed by several PCR analyses for introduction of the hygromycin‐resistance gene‐coding sequence, correct genomic placement of the 5′ and 3′ flanking sequences, and absence of the selected gene sequences (Figures S1b, S2b, and S3b). None of the ∆*AclaeA*, ∆*Acpks*, or ∆*Acgox* mutants showed expression of *AclaeA*, *Acpks*, or *Acgox*, respectively, compared to the WT strain (Figures S1c, S2c, and S3c) when grown in yeast extract sucrose (YES) medium. One of each of the validated ∆*AclaeA*, ∆*Acpks*, and ∆*Acgox* deletion strains were used for the following experiments.

### 2.2 Loss of AcLaeA affects fruit colonization

**FIGURE 1:**
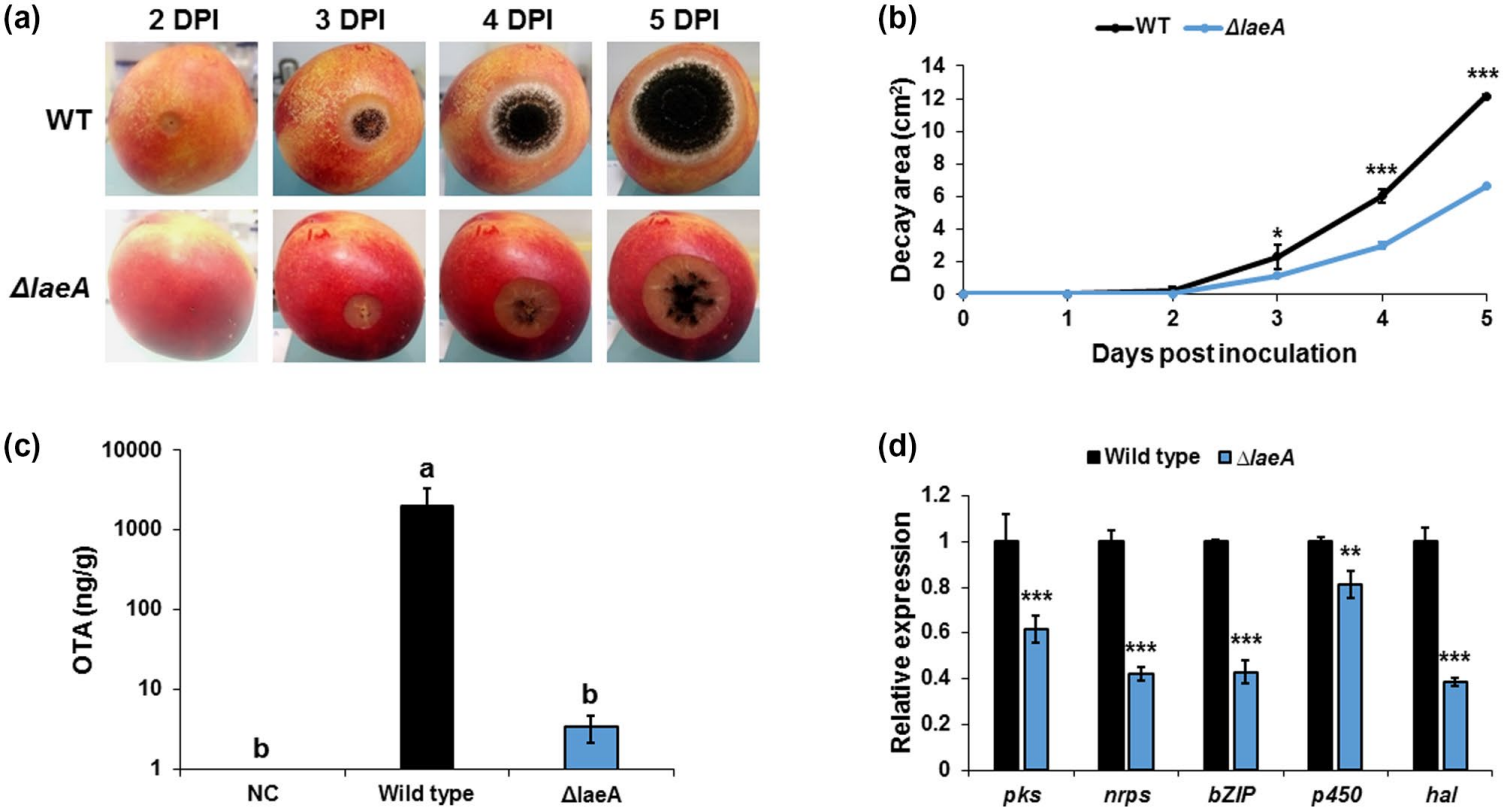
AcLaeA is required for *Aspergillus carbonarius* pathogenicity and ochratoxin A (OTA) production in nectarines. (a) Disease symptoms on nectarine fruits inoculated with conidia of wild‐type (WT) and Δ*AclaeA* strains. (b) Histogram showing the decay area of the rotten tissue on infected nectarines. (c) OTA accumulation in the nectarine tissue and (d) relative expression of OTA cluster genes in WT and Δ*AclaeA* strains. RNA was extracted from infected nectarines at day 5 postinoculation. Relative expression was normalized using *β‐tubulin* as an internal control. Error bars represent standard error of three independent biological replicates. Different letters above the columns indicate statistically significant differences at *p* < .05 as determined using the Tukey's honestly significant difference test. Asterisks denote significant differences between strains at *p* < .05

Virulence of the WT and mutant strains was assessed on nectarines and grapes, which are natural hosts of *A. carbonarius*. Colonization of cv. Sun Snow nectarines and white cv. Zani grape berries by the Δ*AclaeA* strain showed a significant reduction in the rotten colonized area relative to that of the WT strain (Figures 1a and S4a). Five days after inoculation, Δ*AclaeA* showed up to 45% and 21% less colonized area in nectarines and grape berries, respectively, compared to the WT strain (Figures 1b and S4b). Moreover, sporulation in the colonized tissue, which was determined by the area of black developing spores, was significantly reduced in the fruit inoculated with the Δ*AclaeA* mutant compared to the WT strain (Figures 1a and S4a).

Physiological analysis of the Δ*AclaeA* mutant revealed that compared to the WT strain, its growth was not affected on YES synthetic medium at pH 4.0 (Figures S5 and S6a). However, loss of the *laeA* gene resulted in a significant reduction in conidial production as compared to the WT at all examined time points (Figures S5 and S6b). Conidial germination in the Δ*AclaeA* mutant strain was delayed compared to that of the WT, although 100% germination of the former conidia was observed following 8 hr of incubation (Figure S6c). These results indicate that AcLaeA is important for sporulation and conidial germination, and plays a significant role in *A. carbonarius* pathogenicity.

### 2.3 AcLaeA regulates OTA biosynthesis and GLA production

Our findings indicated that inactivation of the *laeA* gene results in almost complete inhibition of OTA production by *A. carbonarius* in YES agar medium (Figure S7). Analysis of OTA accumulation in the colonized nectarine and grape berry tissues 5 days after inoculation revealed a drastic reduction in OTA biosynthesis, from 2,041/534 ng/g by the WT strain to 3.45/17.4 ng/g by the Δ*AclaeA* mutant, respectively (Figures 1c and S4c). Moreover, we investigated the differential expression of all five OTA‐biosynthesis cluster genes (*bZIP* transcription factor, *pks*, nonribosomal peptide synthetase [*nrps*], *p450*, and halogenase [*hal*]) in Δ*AclaeA* and WT strains during fruit infection. The transcript levels of the five genes were down‐regulated in nectarines infected with the Δ*AclaeA* mutant compared to the fruit colonized with the WT strain (Figure 1d). This suggests that AcLaeA is essential for OTA production and is directly involved in regulating transcription of the genes in the OTA biosynthesis pathway.

**FIGURE 2:**
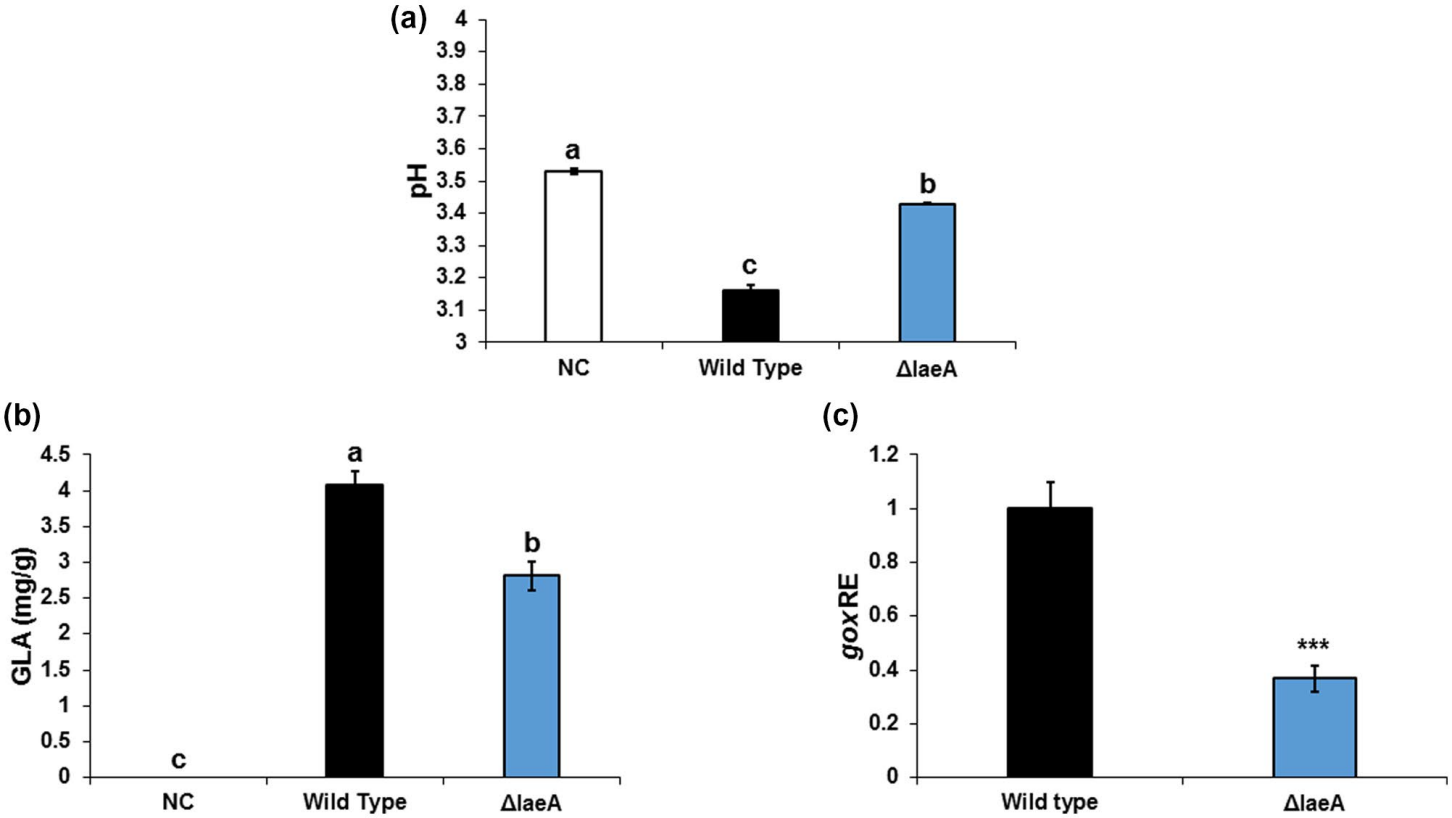
AcLaeA‐associated regulation of gluconic acid (GLA) accumulation in colonized nectarines. (a) The pH of nectarine tissue, (b) GLA accumulation, and (c) *Acgox* relative expression were measured in fruits infected with the wild‐type (WT) and Δ*AclaeA* strains at day 5 postinoculation. Error bars represent standard error of three independent biological replicates. Different letters above the columns indicate statistically significant differences at *p* < .05 as determined using the Tukey's honestly significant difference test. Asterisks denote significant differences between strains at *p* < .05

To gain an understanding of the potential mechanism underlying the reduced pathogenicity of the Δ*AclaeA* strain, the mutant was assessed for some physiological characteristics that have been linked with virulence in this pathogen. We previously reported that one of the factors contributing to the pathogenicity of *A. carbonarius* is its ability to reduce the pH of infected fruit tissue through the production of GLA (Barda et al., 2020; Maor et al., 2017). Indeed, 5 days after inoculation, colonization of nectarine tissue by the *A. carbonarius* WT strain reduced the pH from 3.53 in the healthy part of the fruit to 3.16 in the decayed tissue, whereas the Δ*AcleaA* mutant led to a minimal pH reduction to 3.43 (Figure 2a). Acidification of the rotten tissue by the WT strain was accompanied by the accumulation of 4.06 mg/g of GLA (Figure 2b). In contrast, reduced GLA formation was observed in nectarines colonized by the Δ*AcleaA* strain (2.81 mg/g; Figure 2b). Given that GLA production requires GOX enzyme activity, the observed 3‐fold down‐regulation of *Acgox* gene expression in the Δ*AcleaA* strain could explain the decreased GLA accumulation (Figure 2c). Similarly, during colonization of grape berries, the Δ*AclaeA* mutant showed poor GLA accumulation compared to the WT strain (Figure S4d,e). These results indicate that AcLaeA may also regulate *A. carbonarius* pathogenicity through acidification of the ambient environment by the secretion of organic acids.

### 2.4 OTA accumulation does not contribute to *A. carbonarius* pathogenicity

**FIGURE 3:**
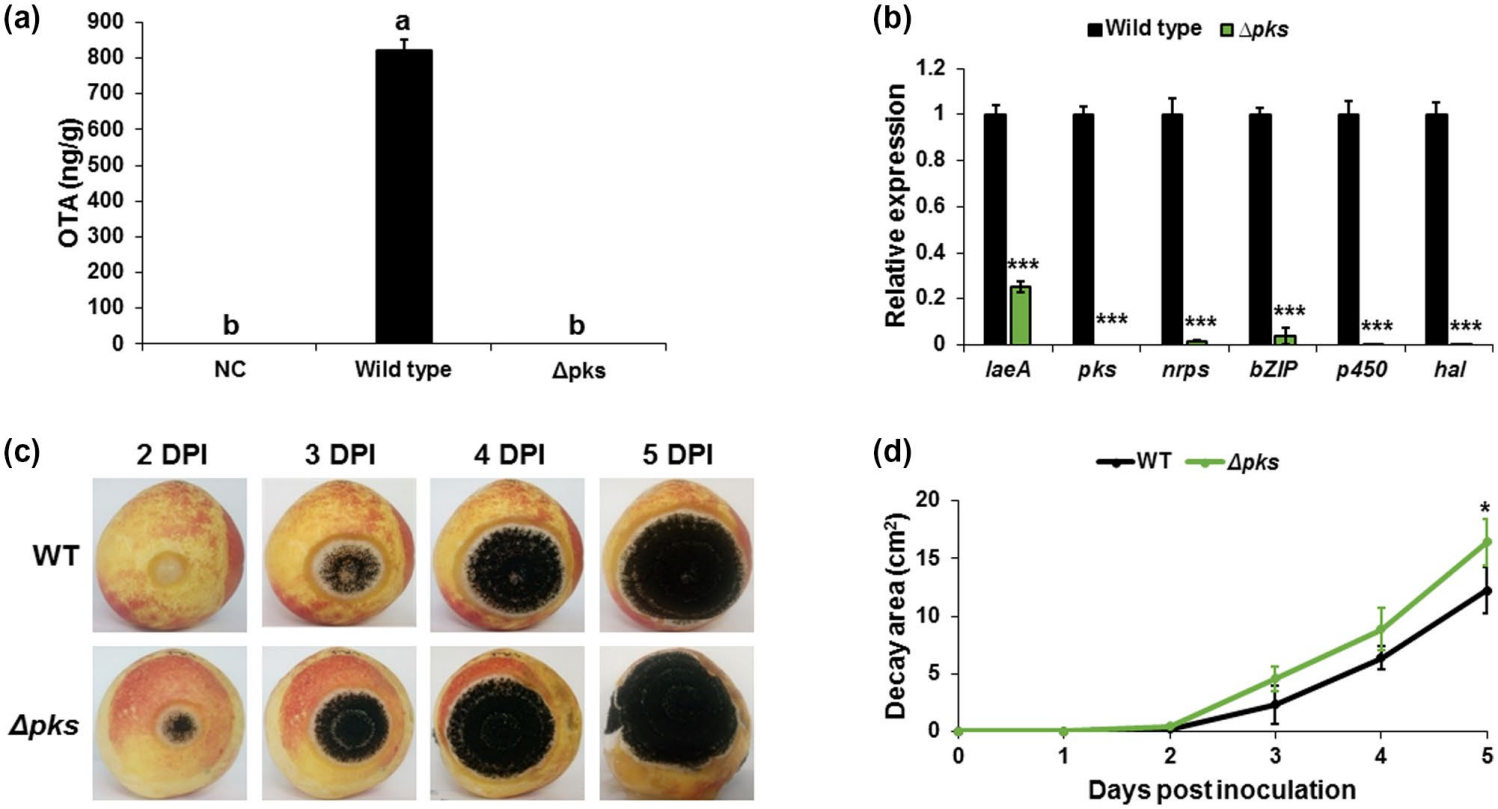
Effect of AcPKS on pathogenicity of *Aspergillus carbonarius* and ochratoxin A (OTA) production in nectarines. (a) OTA accumulation in the nectarine tissue and (b) relative expression of *laeA* and OTA cluster genes in wild‐type (WT) and Δ*Acpks* strains. (c) Disease symptoms on nectarine fruits inoculated with conidia of WT and Δ*Acpks* strains. (d) Histogram showing the decay area of the rotten tissue on infected nectarines. RNA was extracted from infected nectarines at day 5 postinoculation. Relative expression was normalized using *β‐tubulin* as an internal control. Error bars represent standard error of three independent biological replicates. Different letters above the columns indicate statistically significant differences at *p* < .05 as determined using the Tukey's honestly significant difference test. Asterisks denote significant differences between strains at *p* < .05

**FIGURE 4:**
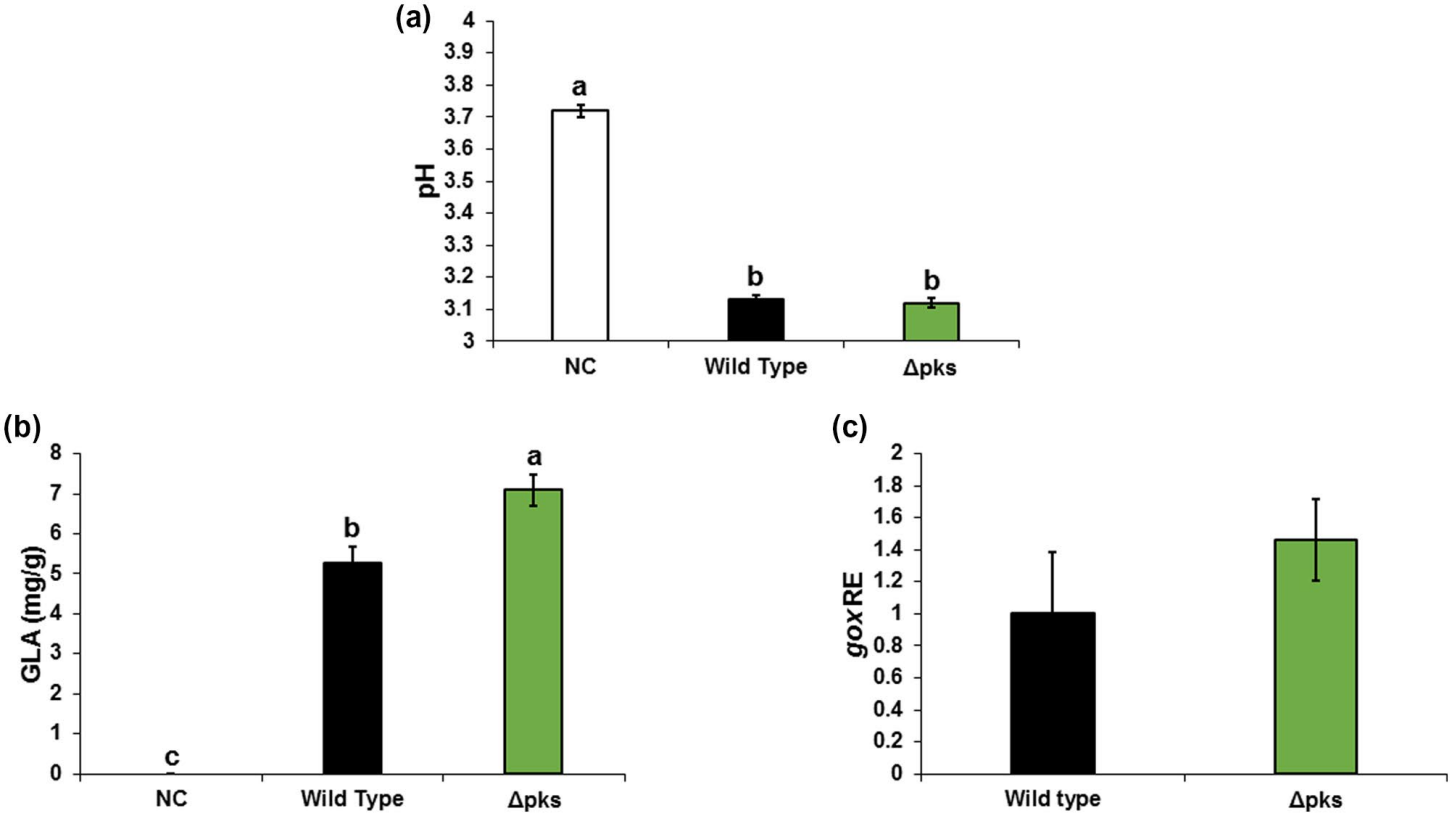
Effect of AcPKS on gluconic acid (GLA) production in colonized nectarines. (a) The pH of nectarine tissues, (b) GLA accumulation, and (c) *Acgox* relative expression were measured in fruits infected with the wild‐type (WT) and Δ*Acpks* strains at day 5 postinoculation. Error bars represent standard error of three independent biological replicates. Different letters above the columns indicate statistically significant differences at *p* < .05 as determined using the Tukey's honestly significant difference test. Asterisks denote significant differences between strains at *p* < .05

To explore the role of OTA in the pathogenicity of *A. carbonarius*, the *Acpks* gene encoding a PKS protein in the OTA cluster was disrupted using the gene‐replacement strategy and *Agrobacterium*‐mediated transformation described in section 2.1 (Figure S2). PKS is considered a key component of the OTA biosynthesis pathway (Moss, 1998). The *Acpks*‐knockout mutant was unable to produce detectable OTA in agar medium, or in colonized fruit, as determined by HPLC analysis (Figures 3a, S8a, and S9a). The loss of *Acpks* resulted in significant down‐regulation of the transcript levels of *laeA* and other genes involved in OTA biosynthesis, including *nrps*, *bZIP*, *p450*, and *hal* (Figure 3b). However, physiological analysis showed no differences in growth, sporulation, or conidial germination patterns between the knockout mutant and the WT strain when grown on YES synthetic medium at pH 4.0 (Figures S5 and S10). Interestingly, deletion of *Acpks* resulted in a slight increase in the rotten colonized area of nectarines and grape berries relative to that of the WT strain (Figures 3c,d and S8b,c). Five days after inoculation, both WT and Δ*Acpks* strains reduced the pH from 3.72 in the healthy part of nectarines to 3.13 and 3.12 in the rotten area, respectively (Figure 4a). The fruit acidification by the Δ*Acpks* mutant was accompanied by higher accumulation of GLA compared to the WT strain (7.08 mg/g versus 5.27 mg/g; Figure 4b), which was positively correlated with increased *gox* expression (Figure 4c). A similar pattern of acidification was observed in the colonized grape berries (Figure S8d,e). Taken together, these results suggest that the fungus’ inability to produce OTA does not affect its pathogenicity.

### 2.5 AcGOX is required for *A. carbonarius* pathogenicity in deciduous fruit

**FIGURE 5:**
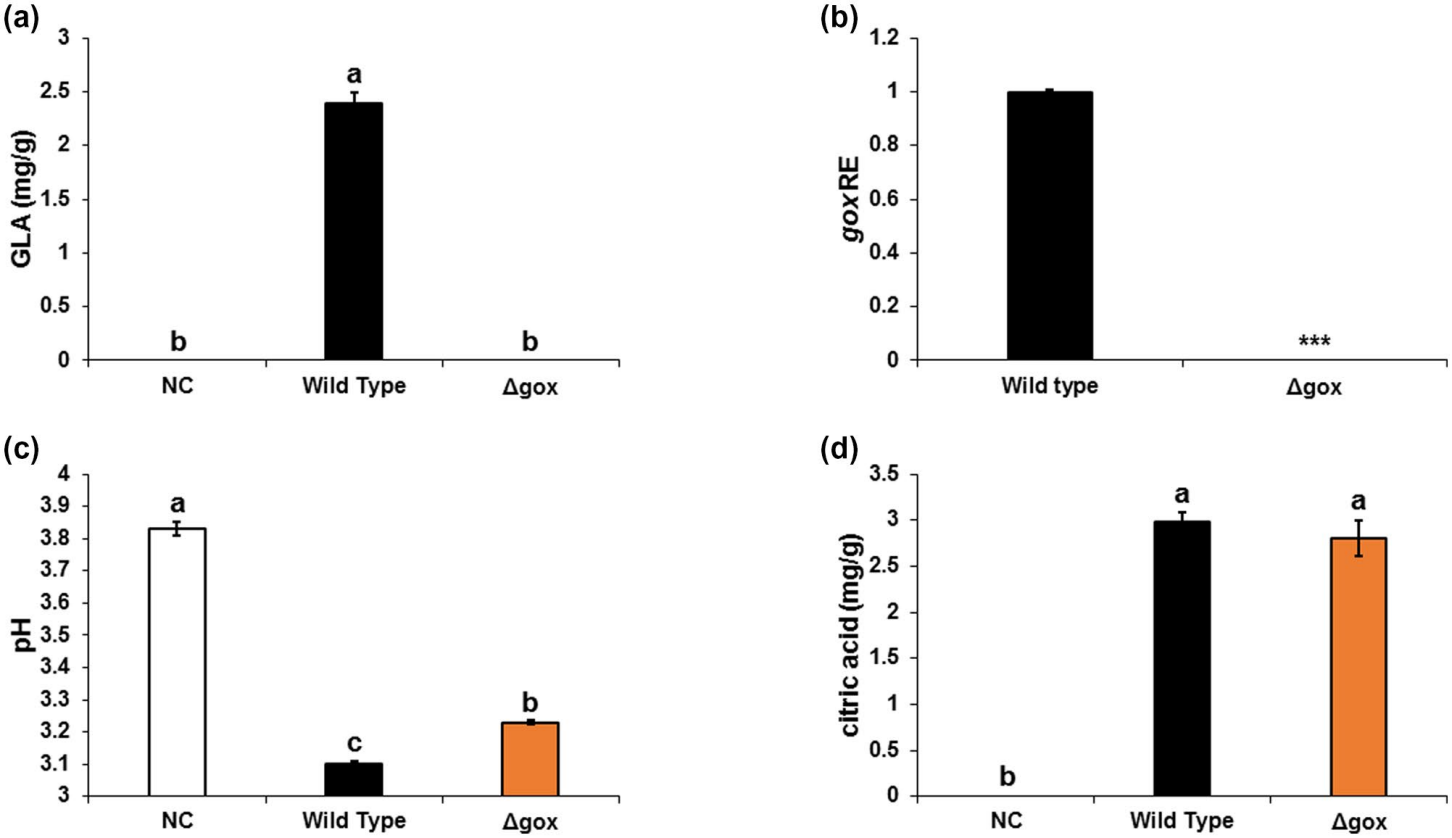
Accumulation of organic acids induced by *Aspergillus carbonarius* Δ*Ac* *gox* mutant in colonized nectarines. (a) gluconic acid (GLA) accumulation, (b) *Acgox* relative expression, (c) pH of nectarine tissues, and (d) accumulation of citric acid were measured in fruits infected with the wild‐type (WT) and Δ*Acgox* strains at day 5 postinoculation. Error bars represent standard error of three independent biological replicates. Different letters above the columns indicate statistically significant differences at *p* < .05 as determined using the Tukey's honestly significant difference test. Asterisks denote significant differences between strains at *p* < .05

**FIGURE 6:**
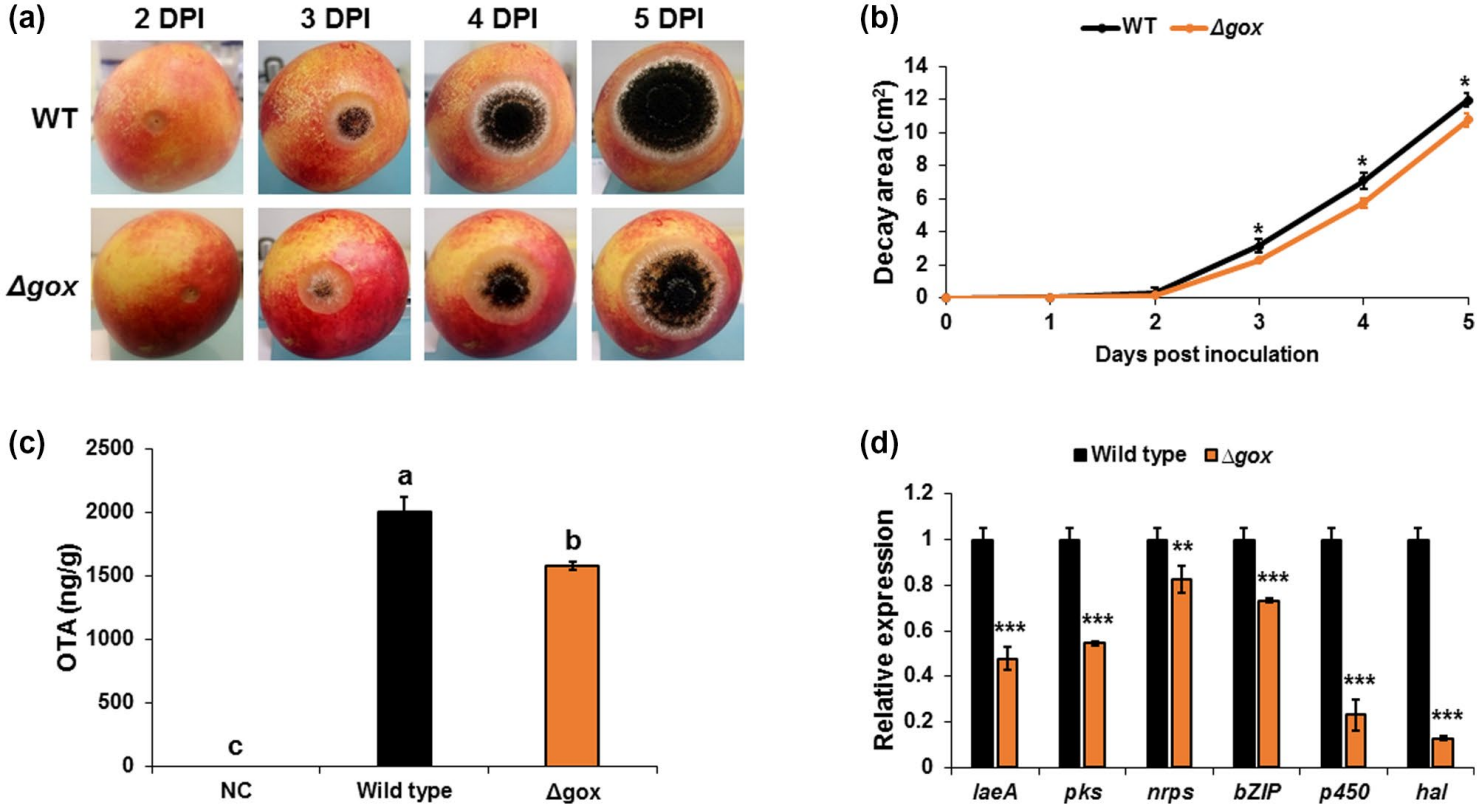
Involvement of glucose oxidase (GOX) in *Aspergillus carbonarius* pathogenicity and ochratoxin A (OTA) production in nectarine fruit. (a) Disease symptoms on nectarine fruits inoculated with conidia of wild‐type (WT) and Δ*Acgox* strains. (b) Histogram showing the decay area of the rotten tissue on infected nectarines. (c) OTA accumulation in the nectarine tissues and (d) relative expression of *laeA* and OTA cluster genes in WT and Δ*Acgox* strains. RNA was extracted from infected nectarines at day 5 postinoculation. Relative expression was normalized using *β‐tubulin* as an internal control. Error bars represent standard error of three independent biological replicates. Different letters above the columns indicate statistically significant differences (*p* < .05) as determined using the Tukey's honestly significant difference test. Asterisks denote significant differences between strains at *p* < .05 (Student's *t* test)

Next, we investigated the role of *Acgox* in *A. carbonarius* pathogenesis. Deletion of *Acgox* completely eliminated GLA formation by *A. carbonarius*, both in vitro and in fruit (Figures 5a, S11a, and S12a). Quantitative reverse transcription PCR (RT‐qPCR) analysis showed lack of *Acgox* expression in the mutant strain, confirming the loss of this gene (Figure 5b). As shown in Figures 6a,b and S11b,c, *Acgox* disruption resulted in a significant reduction of decay development in nectarines and grape berries; the rotten area caused by Δ*Acgox* was about 10% and 18% smaller, respectively, than that caused by the WT strain on day 5 postinoculation. The WT *A. carbonarius* showed a pH reduction from 3.83 in the healthy part of the fruit to 3.1 and 2.46 in the decayed parts of the nectarines and grape berries, respectively (Figures 5c and S11d). This additional acidification of the rotten tissues was accompanied by GLA accumulation of 2.39 and 3.34 mg/g in the nectarines and grape berries, respectively (Figures 5a and S11a). Interestingly, despite its inability to produce GLA, the Δ*Acgox* strain showed a reduction in pH in the inoculated fruit that was similar to that of the WT (Figures 5c and S11d). This phenomenon could be explained by the ability of the Δ*Acgox* strain to accumulate another organic acid, citric acid. In the current work, despite their different levels of pathogenicity, both WT and Δ*Acgox* strains showed accumulation of citric acid in vitro and in vivo (Figures 5d and S12b). Thus, these observations highlight the importance of GOX in *A. carbonarius* virulence in fruit.

Analysis of OTA accumulation in the infected nectarines revealed a moderate reduction in OTA biosynthesis by the Δ*Acgox* strain (1,577 ng/g) compared to the WT strain, which accumulated 2,000 ng OTA/g fruit (Figure 6c). This was confirmed by the down‐regulation of all five genes implicated in OTA biosynthesis, which showed up to 8‐fold reduction in expression in the Δ*Acgox* mutant compared to the WT strain (Figure 6d). Interestingly, *laeA* expression also decreased in the Δ*Acgox* mutant relative to the WT strain (Figure 6d), suggesting an LaeA‐dependent role of GOX in OTA biosynthesis.

### 2.6 AcLaeA and AcGOX differentially modulate the gene expression of cell wall‐degrading enzymes

**FIGURE 7:**
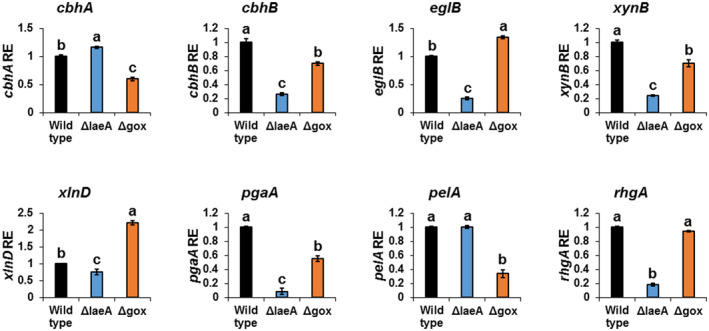
LaeA and GOX regulate the expression of cell‐wall degrading enzymes in *Aspergillus carbonarius*. Relative expression of cell‐wall degrading genes in wild‐type (WT), Δ*AclaeA*, and Δ*Acgox* strains. RNA was extracted from infected nectarines 5 days postinoculation. Error bars represent standard error of three independent biological replicates. Different letters denote significant differences between the strains (*p* < .05)

To gain an additional understanding of the mechanism attenuating the virulence of ∆*AclaeA* and ∆*Acgox* strains in fruit, the transcript levels of eight genes encoding cell wall‐degrading enzymes (de Vries and Visser, 2001) were examined in the deletion mutants during fruit colonization (Figure 7). The expression levels of six genes encoding cellulases (*cbhB*, *eglB*), hemicellulases (*xynB*, *xlnD*), and pectolytic enzymes (polygalacturonase, *pgaA*; rhamnogalacturonase, *rhgA*) were significantly down‐regulated in the ∆*AclaeA* mutant strain compared to the WT (Figure 7). Although five genes encoding cell wall‐degrading enzymes (*cbhA*, *cbhB*, *xynB*, *pgaA*, *pelA*) were down‐regulated in the ∆*Acgox* strain (Figure 7), in general repression of these genes’ expression was not pronounced in the mutant. These results indicate that the genes encoding cell wall‐degrading enzymes are differentially expressed in the mutant strains, and suggest that AcGOX and AcLaeA, in particular, may also modulate *A. carbonarius* virulence through regulation of the enzymes involved in degradation of the host cell wall.

## 3 DISCUSSION

Among the OTA‐producing fungi, *A. carbonarius* is considered the major source of OTA contamination of a variety of fruit, especially grapes and their derived products (Battilani et al., 2006; Perrone et al., 2007). OTA has raised significant public concern worldwide and consequently has impacts on trade, as well as human and animal health. Despite characterization of the OTA biosynthesis gene cluster at the molecular level (Gil‐Serna et al., 2018b), little is known about the regulation of OTA biosynthesis in *A. carbonarius* and its mechanism of fruit colonization. Several factors have been correlated with *A. carbonarius* virulence in fruit, including acidification of the fruit tissue by the fungus (Maor et al., 2017) and the pH regulatory factor PacC (Barda et al., 2020). Those studies demonstrated a clear pattern of pH modulation through secretion of GLA by *A. carbonarius*, which acidifies the ambient environment and induces OTA production both in vitro and in vivo. The accumulation of two fungal metabolites, GLA and OTA, in the colonized tissue raised the question of their contribution, either separately or synergistically, to *A. carbonarius* pathogenicity. Furthermore, we examined the hypothesis that the global regulator of secondary metabolism, LaeA, which is one of the major virulence factors in several ascomycete fungi, plays an important role in *A. carbonarius* OTA synthesis and virulence on fruit.

A common putative cluster of genes involved in OTA biosynthesis has been recently described in the main *Aspergillus* (including *A. carbonarius*) and *Penicillium* OTA‐producing species (Gil‐Serna et al., 2018b). However, no details have been published on the mechanism activating this cluster. Given the importance of LaeA in the regulation of secondary metabolites, we analysed the functional role of this transcriptional factor in OTA biosynthesis by deleting *laeA* in *A. carbonarius*. Functional analysis of the Δ*AclaeA* mutant strain in colonized fruit showed significant down‐regulation of all five genes in the OTA cluster, accompanied by a marked reduction in OTA production (Figures 1c,d and S4e), suggesting direct regulation of OTA biosynthesis by LaeA. Interestingly, concurrent with the decrease in OTA accumulation, the severity of the disease caused by the Δ*AclaeA* strain declined by up to 45% compared to that caused by the WT in colonized fruit. Given that GLA formation is associated with *A. carbonarius* virulence (Barda et al., 2020; Maor et al., 2017), a 3‐fold reduction in *Acgox* expression accompanied by reduced GLA accumulation in the Δ*AclaeA* mutant may also have contributed to the reduction in this strain's virulence, suggesting that AcLaeA may influence fungal virulence by mediating a virulence factor, AcGOX. The question that arose here was whether the reduced pathogenicity of the ∆*Ac* *laeA* mutant is due to the decline in OTA accumulation or repressed tissue acidification.

The biological role of fungal secondary metabolites has not been elucidated, but in some studies mycotoxins have been shown to contribute to the pathogenicity of several fungi. *Fusarium*‐produced toxins, such as deoxynivalenol and nivalenol, have been shown to be involved in the virulence of *Fusarium graminearum* toward wheat, maize, and barley (Boddu et al., 2007; Maier et al., 2006; Proctor et al., 2002). Several studies have demonstrated OTA phytotoxicity on the plant model organism *Arabidopsis thaliana*, and suggested that this toxin plays a role in the aetiology of plant diseases (Peng et al., 2010; Wang et al., 2012). Those studies indicated that OTA causes a rapid hypersensitive response and induces necrotic lesions in host leaf tissues. Exposure to OTA stimulated an oxidative burst in the plant, which resulted in a significant increase in reactive oxygen species and enhanced antioxidant enzyme defence responses, leading to plant cell death (Peng et al., 2010; Wang et al., 2012). However, the results obtained from the current work clearly show that OTA is not associated with fungal disease development in fruit. The deletion of *Acpks* in *A. carbonarius*, a key gene in the OTA cluster involved in OTA biosynthesis, did not affect fungal virulence on the colonized fruit (Figures 3 and S8). Similar findings have been reported in recently published studies, where deletion of *patL* and *patK* genes, encoding specific regulatory factors of the patulin‐biosynthesis pathway and PKS, respectively, did not affect *P. expansum* virulence and demonstrated that patulin is not required by the fungus to infect apples (Ballester et al., 2015; Li et al., 2015). Interestingly, a study using a *patL*‐knockout mutant also demonstrated that patulin is not required by *P. expansum* to colonize apples, but acts as a cultivar‐dependent aggressiveness factor (which describes the severity of disease) (Snini et al., 2016). Given that the experiments in the current study on the involvement of OTA in the pathogenicity of *A. carbonarius* were conducted only on specific nectarine (Sun Snow) and grape (Zani) varieties, it is possible that OTA acts as a virulence factor in a host‐dependent manner.

Our previous studies indicated that GOX, which is essential for GLA production and acidification during fruit colonization, is important for *A. carbonarius* pathogenicity (Barda et al., 2020; Maor et al., 2017). GOX has been reported as a virulence factor in *P. expansum* (Barad et al., 2012; Chen et al., 2018). Down‐regulated expression of the GOX‐encoding gene *gox2* reduces the infection process in the fruit, resulting in a decrease in disease incidence of *P. expansum*. To confirm involvement of GLA in *A. carbonarius* virulence, the *Acgox* gene was disrupted. Deletion of *Acgox* resulted in complete inhibition of GLA formation and led to a reduction in virulence toward nectarine and grape fruit (Figures 6 and S11), further indicating that GOX is a virulence factor of *A. carbonarius*. It should be noted that the inoculated fruit were acidified to a similar extent by both the Δ*Acgox* mutant and the WT strain. The acidification by the former could be explained by its ability to produce a relatively high amount of citric acid both in vitro and in vivo (Figures 5 and S12). Our findings are consistent with those of Yang et al. (2014), who reported that deletion of *gox* in *A. carbonarius* changes the carbon flux toward other organic acids. As a consequence of the gene deletion, GLA accumulation was completely inhibited and increased amounts of other organic acids, including citric acid, were observed in the Δ*Ac* *gox* mutant. In this regard, it is worth mentioning that in our study, despite the lack of GLA production, the reduction in pathogenicity of the *A. carbonarius* Δ*gox* mutant was minor relative to that observed with the Δ*AclaeA* mutant.

Cell wall‐degrading enzymes are known to contribute to the pathogenicity of fungal plant pathogens (ten Have et al., 2002). A number of transcription factors regulating fungal virulence and secondary metabolism in filamentous fungi have been identified to control the expression of genes encoding plant cell wall‐degrading enzymes. The influence of the carbon catabolite repressor protein (CreA) on the plant cell wall‐degrading enzymes of several *Aspergillus* species has been extensively studied. In those species, CreA has been reported to act as a negative regulator of genes encoding arabinases, several endoxylanases, and pectinolytic enzymes (Bussink et al., 1991; Kester et al., 1996; Orejas et al., 1999; Ruijter et al., 1997). *creA* mutant strains of *A. nidulans* and *Aspergillus niger* displayed (partially) derepressed phenotypes (Ruijter et al., 1997; Shroff et al., 1997). The pH‐regulatory protein PacC has also been shown to be involved in controlling genes encoding cell wall‐degrading enzymes. Alkaline‐ and acidic‐mimicking *A. nidulans* *pacC* mutants showed that the major arabinofuranosidase‐encoding gene, *abfB*, and two genes encoding xylanases, *xlnA* and *xlnB*, are regulated by the PacC transcription factor (Gielkens et al., 1999; MacCabe et al., 1998). A recent study has suggested that PacC contributes to the pathogenesis of *A. carbonarius* through regulation of cell wall‐degrading enzymes during fruit colonization (Barda et al., 2020). In the present study, LaeA positively regulated the expression of most of the tested genes encoding cell wall‐degrading enzymes in *A. carbonarius* (Figure 7). These results were in agreement with previous reports on the interaction of an endophytic fungus *Epichloë festucae* with perennial ryegrass, which suggested a regulatory role for LaeA in the expression of genes for plant cell wall degradation (Rahnama et al., 2019, 2020). We assume that the deletion of *AclaeA* could affect *A. carbonarius* pathogenicity through down‐regulation of the cell wall‐degrading enzymes, including cellulases, hemicellulases, and pectolytic enzymes. Another key factor contributing to fungal pathogenicity in colonized fruit, GOX, has been reported to induce transcript levels of *pmpg1* (encoding endopolygalacturonase) in *Phomopsis mangiferae* during colonization of grapes (Davidzon et al., 2010). In the current work, using the ∆*Acgox* mutant strain, a number of cell wall‐degrading enzyme‐encoding genes were shown to be regulated by GOX, although we found evidence of weaker repression of these genes in ∆*Acgox* compared to the ∆*AcleaA*‐knockout strain (Figure 7).

The results of this study strongly suggest that the global regulator of secondary metabolites, LaeA, contributes to *A. carbonarius* pathogenicity. We showed that *Acgox*, which is essential for GLA production and acidification during fruit colonization, is significantly down‐regulated in the Δ*AclaeA* mutant, suggesting that LaeA governs *A. carbonarius* pathogenicity through regulation of the expression of *Acgox*. Deletion of the *Acgox* gene in *A. carbonarius* led to a reduction in virulence toward grapes and nectarine fruit, further indicating that GOX is a virulence factor of *A. carbonarius* and that its expression is regulated by LaeA. It is also clear from the present data that, similar to other mycotoxins assessed to date, LaeA is a positive regulator of OTA biosynthesis both in vitro and during fruit colonization. However, our study clearly demonstrates that OTA accumulation does not contribute to the pathogenicity of *A. carbonarius* in grapes and nectarine fruit. The Δ*Acpks* mutant, which is unable to produce OTA, induced disease in fruit cultivars in a manner similar to the WT strain. Although some progress has been achieved toward understanding the mechanisms governing pathogenicity and OTA biosynthesis in *A. carbonarius*, several questions remain unanswered. Transcriptomic analysis of the Δ*AclaeA* and Δ*Acgox* mutants during infection may provide a clearer understanding of the genetic and molecular regulatory mechanisms involved in secondary metabolism and fungal virulence in *A. carbonarius*. This study was important in improving our basic understanding of the pathogenic mechanism of *A. carbonarius* and mycotoxin accumulation during fruit colonization. Regulation of the processes modulated by the transcription factor LaeA is of extreme importance for *Aspergillus* virulence; the regulation of secondary metabolism during the secretion of organic acids could serve as a model system for understanding the pH‐regulating processes in other fungal pathogens as well. Modulation of fruit tissue pH during postharvest disease development might enable the design of control measures that focus on alleviating the consequences of pathogen‐secreted pH modifiers.

## 4 EXPERIMENTAL PROCEDURES

### 4.1 Fungal strains and culture conditions

*A. carbonarius* isolate NRRL 368 was obtained from the USDA Agricultural Research Service Culture Collection. This WT strain was used to generate all derivative mutants described in the following section. Cultures were grown at 28 °C in the dark and maintained on potato dextrose agar (PDA; Becton Dickinson). Conidia were harvested using 5 ml of sterile distilled water and filtered through a 40‐µm cell strainer (Biologix) to remove hyphae. Cells were visualized with a BH‐2 series microscope (Olympus) and adjusted to the indicated concentrations using a haemocytometer.

### 4.2 Construction of plasmids and knockout strains

The *AclaeA*, *Acpks*, and *Acgox* deletion vectors were generated using hygromycin B as a selectable marker. For each gene‐replacement plasmid, the target gene's genomic flanking regions were PCR‐amplified using specific primer pairs that incorporated a single 2‐deoxyuridine nucleoside near the 5′ ends. Both DNA fragments and the predigested pRFHU2 binary vector (Frandsen et al., 2008) were mixed together and treated with the USER enzyme (New England Biolabs) to obtain the deletion vector. An aliquot of the mixture was used directly for chemical transformation of high‐efficiency *Escherichia coli* DH5α cells (New England Biolabs) without prior ligation. Kanamycin‐resistant transformants were screened by PCR for validation of proper fusion events. The plasmid was then introduced into electrocompetent *A. tumefaciens* AGL‐1 cells.

A single colony of *A. tumefaciens* AGL‐1 carrying the desired plasmid (pRFHU2‐AclaeA, pRFHU2‐Acpks, or pRFHU2‐Acgox) was used to inoculate a starter culture and incubated for 24 hr. Bacterial cells were centrifuged, washed with induction medium (10 mM K_2_HPO_4_, 10 mM KH_2_PO_4_, 2.5 mM NaCl, 2 mM MgSO_4_, 0.6 mM CaCl_2_, 9 μM FeSO_4_, 4 mM (NH_4_)_2_SO_4_, 10 mM glucose, 40 mM MES pH 5.3, 0.5% glycerol), and diluted in the same medium amended with 200 μM acetosyringone (IMAS) to an OD_600_ of 0.15. Cells were grown at 28 °C and 200 rpm until they reached an OD_600_ of 0.75. Equal volumes of IMAS‐induced bacterial culture and a conidial suspension of *A. carbonarius* (10^6^ conidia/ml) were mixed and spread onto Whatman filter papers, which were placed on agar plates containing the cocultivation medium (same as IMAS, but containing 5 mM instead of 10 mM glucose). After cocultivation at 28 °C for 48 hr, the filter papers were transferred to PDA plates containing hygromycin B (100 μg/ml) as the selection agent for fungal transformants and cefotaxime (200 μg/ml) to inhibit growth of *A. tumefaciens* cells. Hygromycin‐resistant colonies appeared after 3–4 days of incubation at 28 °C. Successful disruptions of the target genes were confirmed by PCR analysis of the transformants. All primers used to create and confirm the mutant strains are listed in Table S1.

### 4.3 Physiological analysis of *A. carbonarius* strains

Both vegetative growth and sporulation of each of the knockout and WT strains were assessed on YES agar (20 g Bacto yeast extract, 150 g sucrose, 15 g Bacto agar per litre) adjusted with HCl to pH 4, whereas germination assays were performed in YES broth (20 g Bacto yeast extract and 150 g sucrose per litre) adjusted to pH 4.

To measure the radial mycelial growth, agar plates were point‐inoculated with 10^2^ spores of each strain and incubated at 28 °C. Radial growth was monitored by diameter measurements on a daily basis up to 7 days using three replicate plates per strain.

To quantify total conidia produced by the various strains, agar plates containing 10^5^ spores were incubated at 28 °C in the dark. To accurately count conidia, two 1‐cm plugs from each plate were homogenized in 3 ml of 0.01% Tween 20 in water, diluted, and counted with a haemocytometer. Conidial production was quantified starting at 24 hr postinoculation using three replicate plates per strain.

Germination assays were performed in sterile 24‐well culture plates (SPL Life Sciences). The spore concentration of all strains was adjusted to 10^4^ spores/ml in the medium, and 0.5 ml of each spore suspension was distributed into three replicate wells. Time‐course microscopy was carried out over 24 hr at 28 °C using an Eclipse T*i* inverted microscope (Nikon). The germination rate was monitored regularly by capturing images of each well at 1‐hr intervals. The number of spore germlings was counted for each strain and recorded. The percentage of germinated spores was plotted against time, and the germination rates were determined.

### 4.4 OTA and organic acid accumulation

A 100 µl suspension of 10^6^ fungal spores/ml was inoculated onto 55‐mm Petri dishes containing 10 ml of YES agar adjusted to pH 4. The plates were incubated at 28 °C in the dark for up to 10 days as needed for sample collection.

To assess organic acid production, five 1‐cm diameter discs of agar were placed in 5 ml of sterilized water and crushed to homogeneity. A 1‐ml aliquot of the solution was sampled in a 1.5‐ml microcentrifuge tube and centrifuged for 10 min at 20,800 × g. The supernatant was taken for GLA and citric acid analyses using test kits that apply enzymatic methods for the specific measurement of total d‐gluconic acid and citric acid contents (Megazyme) according to the manufacturer's instructions. The final pH was measured directly in the agar cultures with a double‐pore slim electrode connected to a Sartorius PB‐11 Basic Meter.

To evaluate OTA levels, five 1‐cm diameter discs of agar were added to 1.7 ml of HPLC‐grade methanol (Bio‐Lab) and crushed to homogeneity. OTA was extracted by shaking for 30 min at 150 rpm on an orbital shaker and centrifuged for 10 min at 20,800 × g. The supernatant was filtered through a 0.22‐µm PTFE syringe filter (Agela Technologies) and kept at −20 °C prior to HPLC analysis. OTA was quantitatively analysed by injection of 20 µl into a reversed‐phase UHPLC system (Waters ACQUITY Arc, FTN‐R). The mobile phase consisted of acetonitrile:water:acetic acid (99:99:2, vol/vol/vol) at 0.5 ml/min through a Kinetex 2.6 µm XB‐C18 (100 × 2.1 mm) with a security guard C18 column (4 × 2 mm) (Phenomenex). The column temperature was maintained at 30 °C. The OTA peak was detected with a fluorescence detector (excitation at 330 nm and emission at 450 nm) and quantified by comparing with a calibration curve of the standard mycotoxin (Fermentek).

### 4.5 Colonization and virulence assessment of *A. carbonarius* strains

Zani seedless grapes and Sun Snow nectarines were obtained from a local supermarket. Fruit surfaces were sterilized in 1% sodium hypochlorite solution for 1 min and immediately rinsed in sterile distilled water. A 10‐µl spore suspension containing 10^6^ spores/ml of either the WT or the knockout strains was injected directly into the sterilized fruit at 2 mm depth. Following inoculation, the fruit were incubated under high humidity at 28 °C for 2–7 days as needed for symptom monitoring and sample collection. The area of the necrotic lesions was recorded daily. The pH of nectarine and grape berry tissues was measured by inserting the double‐pore slim electrode connected to the Sartorius PB‐11 Basic Meter directly into the tested area.

To analyse the GLA content of the inoculated fruit, 1.7 g of the macerated necrotic area was homogenized in 5 ml sterilized water. A 1‐ml aliquot of the solution was sampled in a 1.5‐ml microcentrifuge tube and centrifuged for 10 min at 20,800 × g, and the amount of GLA produced was measured as described above (section 4.4).

For OTA analysis in colonized grapes and nectarines, 1.7 g of the macerated necrotic area was homogenized in 1.7 ml of HPLC‐grade methanol. Then, OTA was quantitatively analysed as described above (section 4.4).

### 4.6 RNA isolation and RT‐qPCR analysis of gene expression

Mycelia grown on agar plates were harvested on day 5 postinoculation, frozen in liquid nitrogen, lyophilized for 24 hr, and kept at −80 °C until use. In colonized nectarines, mycelium‐containing exocarp (peel) was removed on day 5 postinfection, frozen in liquid nitrogen, lyophilized for 24 hr, and kept at −80 °C prior to RNA extraction. Total RNA was extracted from 100 mg of lyophilized tissue of the selected samples using the Hybrid‐R RNA isolation kit (GeneAll) according to the manufacturer's protocol. The DNase and reverse‐transcription reactions were performed on 1 µg of total RNA with the Maxima First‐Strand cDNA Synthesis Kit (Thermo Scientific) according to the manufacturer's instructions. The cDNA samples were diluted 1:10 (vol/vol) with ultrapure water. The RT‐qPCR was performed using Fast SYBR green Master Mix (Applied Biosystems) in a StepOnePlus Real‐Time PCR System (Applied Biosystems). The PCR conditions were as follows: 95 °C for 20 s, followed by 40 cycles of 95 °C for 3 s and 60 °C for 20 s. The expression of the genes analysed in this work was quantified using RT‐qPCR optimization protocols (standard curve, amplification efficiency, correlation coefficient, and dissociation curve) that were previously developed by Gil‐Serna et al. (2018b). The samples were normalized using *β‐tubulin* as the endogenous control and the relative expression levels were measured using the 2^(−ΔΔ^ *^C^* ^t)^ analysis method. Results were analysed with StepOne v. 2.3 software. The primers used for the RT‐qPCR analyses are listed in Table S2.

### 4.7 Statistical analysis

Student's *t* test was performed when data were normally distributed and the sample variances were equal. For multiple comparisons, one‐way analysis of variance (ANOVA) was performed when the equal variance test was passed. If one‐way ANOVA reported a *p* value of <.05, further analyses were performed using Tukey's single‐step honestly significant difference test to determine significant differences between the strains. All experiments described here are representative of at least three independent experiments with the same patterns of results.

## Supporting information


**FIGURE S1** Generation of Δ*AclaeA* knockout mutant. (a) Schematic representation to scale of the deletion of *laeA* gene. The gene replacement vector pRFHU2‐AclaeA was constructed by cloning the 5′ and 3′ flanking regions on each side of the hygromycin resistance gene *hph*. (b) Verification of the positive transformants by PCR analysis. Primer pairs L‐f1 × L‐r1 and H‐f1 × H‐r1 were used to verify the removal of the *AclaeA* ORF and the acquisition of the *hph* ORF in the deletant strain, respectively. A fragment of nonspecific *pacC* gene was amplified to verify the integrity of the Δ*laeA* and wild‐type genomic DNA. Primers for amplification of the flanking fragments and verification of the positive transformants are listed in Table S1. (c) Quantitative reverse transcription PCR analysis of *laeA* expression in the wild‐type strain and Δ*laeA* mutants

**FIGURE S2** Generation of *ΔAcpks* knockout mutant. (a) Schematic representation to scale of the deletion of *pks* gene. The gene replacement vector pRFHU2‐Acpks was constructed by cloning the 5′ and 3′ flanking regions on each side of the hygromycin resistance gene *hph*. (b) Verification of the positive transformants by PCR analysis. Primer pairs P‐f1 × P‐r1 and H‐f1 × H‐r1 were used to verify the removal of the *Acpks* ORF and the acquisition of the *hph* ORF in the deletant strain, respectively. A fragment of nonspecific *pacC* gene was amplified to verify the integrity of the Δ*pks* and wild‐type genomic DNA. Primers for amplification of the flanking fragments and verification of the positive transformants are listed in Table S1. (c) Quantitative reverse transcription PCR analysis of *pks* expression in the wild‐type strain and Δ*pks* mutants

**FIGURE S3** Generation of *ΔAcgox* knockout mutant. (a) Schematic representation to scale of the deletion of *gox* gene. The gene replacement vector pRFHU2‐Acgox was constructed by cloning the 5′ and 3′ flanking regions on each side of the hygromycin resistance gene *hph*. (b) Verification of the positive transformants by PCR analysis. Primer pairs G‐f1 × G‐r1 and H‐f1 × H‐r1 were used to verify the removal of the *Acgox* ORF and the acquisition of the *hph* ORF in the deletant strain, respectively. A fragment of nonspecific *pacC* gene was amplified to verify the integrity of the Δ*gox* and wild‐type genomic DNA. Primers for amplification of the flanking fragments and verification of the positive transformants are listed in Table S1. (c) Quantitative reverse transcription PCR analysis of *gox* expression in the wild‐type strain and Δ*gox* mutants

**FIGURE S4** Effect of LaeA on *Aspergillus carbonarius* virulence and OTA production in grapes. Growth development of the wild type and *∆laeA* strains of *A. carbonarius* on freshly harvested grape berries (a, b). OTA accumulation, pH changes and GLA production in grape berries (c–e). Error bars represent the standard error of three independent biological replicates. Different letters above the columns indicate statistically significant differences (*p* < .05) as determined using the Tukey’s honestly significant difference test. Asterisks denote significant differences between strains at *p* < .05 (Student’s *t* test)

**FIGURE S5** Growth phenotype of the wild type, Δ*laeA*, Δ*gox*, and Δ*pks* mutant strains of *Aspergillus carbonarius* in YES medium under pH 4 at 28 °C

**FIGURE S6** Physiological analyses of the wild type (WT) and *∆laeA* strains of *Aspergillus carbonarius*. (a) Radial growth of the WT and *∆laeA* strains on solid YES medium at 28 °C at pH 4. (b) Conidiation of the WT and *∆laeA* strains on solid YES medium at pH 4. (c) Germination rates in the WT and *∆laeA* strains were assessed in static YES broth at 28 °C at pH 4. Error bars represent the standard error of the mean (*SEM*) across three independent replicates. Asterisks denote significant differences between strains at *p* < .05 (Student’s *t* test)

**FIGURE S7** Effect of LaeA on OTA and GLA production in *Aspergillus carbonarius*: (a) OTA accumulation, (b) OTA cluster gene expression, (c) GLA accumulation, and (d) *gox* expression by the wild‐type and ∆*laeA* strains of *A. carbonarius* when grown on YES medium under pH 4 at 28 °C. Asterisks denote significant differences between strains (*p* < .05)

**FIGURE S8** Effect of PKS on *Aspergillus carbonarius* virulence and OTA synthesis in grapes: (a) OTA accumulation and (b, c) growth development of the wild type and *∆pks* strains of *A. carbonarius* on freshly harvested grapes. (d) pH changes and (e) GLA accumulation in grape berries. Error bars represent the standard error of the mean (*SEM*) across three independent replicates. Different letters above the columns indicate statistically significant differences (*p* < .05) as determined using the Tukey’s honestly significant difference test

**FIGURE S9** Effect of PKS on OTA and GLA production in *Aspergillus carbonarius* in vitro: (a) OTA accumulation, (b) *laeA* and OTA cluster gene expression, (c) GLA accumulation, and (d) *gox* gene expression by the wild type and *∆pks* strains of *A. carbonarius* when grown in YES medium under pH 4 at 28 °C. Asterisks denote significant differences between strains (*p* < .05)

**FIGURE S10** Physiological analyses of the wild type (WT) and *∆pks* strains of *Aspergillus carbonarius*. (a) Radial growth of the WT and *∆pks* strains on solid YES medium at 28 °C at pH 4. (b) Conidiation of the WT and *∆pks* strains on solid YES medium at pH 4. (c) Germination rates in the WT and *∆pks* strains were assessed in static YES broth at 28 °C at pH 4. Error bars represent the standard error of the mean (*SEM*) across three independent replicates. Asterisks denote significant differences between strains at *p* < .05 (Student’s *t* test)

**FIGURE S11** Involvement of GOX in *Aspergillus carbonarius* virulence and OTA biosynthesis in grapes: (a) GLA production and (b, c) growth development of the wild type and *∆gox* strains of *A. carbonarius* on freshly harvested grapes. (d) pH changes and (e) OTA accumulation in grape berries. Error bars represent the standard error of the mean (*SEM*) across three independent replicates. Different letters above the columns indicate statistically significant differences (*p* < .05) as determined using the Tukey’s honestly significant difference test. Asterisks denote significant differences between strains at *p* < .05 (Student’s *t* test)

**FIGURE S12** The effect of *Acgox* gene deletion on the production of organic acids by *Aspergillus carbonarius*: (a) GLA production and (b) citric acid accumulation by the WT and *∆gox* strains of *A. carbonarius* grown on YES culture medium under pH 4.0 at 28 °C. Asterisks denote significant differences between strains at *p* < .05 (Student's *t* test)

**TABLE S1** List of primers used in the study to create and confirm the mutant strains

**TABLE S2** Primer sequences used for quantitative reverse transcription PCR analysis

## Data Availability

The data that support the findings of this study are available from the corresponding author upon reasonable request.
