# Supplementary material for: Functional roles of LaeA, polyketide synthase, and glucose oxidase in the regulation of ochratoxin A biosynthesis and virulence in Aspergillus carbonarius
Source: Mol Plant Pathol. 2020 Nov 10;22(1):117–29. doi: 10.1111/mpp.13013 (PMC7749749; doi:10.1111/mpp.13013)
Supplement: Supplementary file 1 — FIGURE S1 Generation of ΔAclaeA knockout mutant. (a) Schematic representation to scale of the deletion of laeA gene. The gene replacement vector pRFHU2‐AclaeA was constructed by cloning the 5′ and 3′ flanking regions on each side of the hygromycin resistance gene hph. (b) Verification of the positive transformants by PCR analysis. Primer pairs L‐f1 × L‐r1 and H‐f1 × H‐r1 were used to verify the removal of the AclaeA ORF and the acquisition of the hph ORF in the deletant strain, respectively. A fragment of nonspecific pacC gene was amplified to verify the integrity of the ΔlaeA and wild‐type genomic DNA. Primers for amplification of the flanking fragments and verification of the positive transformants are listed in Table S1. (c) Quantitative reverse transcription PCR analysis of laeA expression in the wild‐type strain and ΔlaeA mutants [file MPP-22-117-s001.docx]

**Figure S1. Generation of *ΔAclaeA* knockout mutant.** **(a)** Schematic representation to scale of the deletion of *laeA* gene. The gene replacement vector pRFHU2-AclaeA was constructed by cloning the 5' and 3' flanking regions on each side of the hygromycin resistance gene *hph*. **(b)** Verification of the positive transformants by PCR analysis. Primer pairs L-f1 x L-r1 and H-f1 x H-r1 were used to verify the removal of the *AclaeA* ORF and the acquisition of the *hph* ORF in the deletant strain respectively. A fragment of non-specific *pacC* gene was amplified in order to verify the integrity of the *ΔlaeA* and WT gDNA. Primers for amplification of the flanking fragments and verification of the positive transformants are listed in supplementary table S1. **(c)** qPCR analysis of *laeA* expression in the WT strain and *ΔlaeA* mutants.
