# Supplementary material for: Functional roles of LaeA, polyketide synthase, and glucose oxidase in the regulation of ochratoxin A biosynthesis and virulence in Aspergillus carbonarius
Source: Mol Plant Pathol. 2020 Nov 10;22(1):117–29. doi: 10.1111/mpp.13013 (PMC7749749; doi:10.1111/mpp.13013)
Supplement: Supplementary file 4 — FIGURE S4 Effect of LaeA on Aspergillus carbonarius virulence and OTA production in grapes. Growth development of the wild type and ∆laeA strains of A. carbonarius on freshly harvested grape berries (a, b). OTA accumulation, pH changes and GLA production in grape berries (c–e). Error bars represent the standard error of three independent biological replicates. Different letters above the columns indicate statistically significant differences (p < .05) as determined using the Tukey’s honestly significant difference test. Asterisks denote significant differences between strains at p < .05 (Student’s t test) [file MPP-22-117-s004.docx]

**Figure S4. Effect of LaeA on *A. carbonarius* virulence and OTA production in grapes.** Growth development of the WT and *∆laeA* strains of *A. carbonarius* on freshly harvested grape berries **(a, b)**. OTA accumulation, pH changes and GLA production in grape berries **(c, d, e)**. Error bars represent standard error of three independent biological replicates. Different letters above the columns indicate statistically significant differences (*p*<0.05), as determined using the Tukey's honest significant difference test. Asterisks denote significant differences between strains at *p*<0.05 (Student's *t* test).
