# Supplementary material for: Functional roles of LaeA, polyketide synthase, and glucose oxidase in the regulation of ochratoxin A biosynthesis and virulence in Aspergillus carbonarius
Source: Mol Plant Pathol. 2020 Nov 10;22(1):117–29. doi: 10.1111/mpp.13013 (PMC7749749; doi:10.1111/mpp.13013)
Supplement: Supplementary file 5 — FIGURE S5 Growth phenotype of the wild type, ΔlaeA, Δgox, and Δpks mutant strains of Aspergillus carbonarius in YES medium under pH 4 at 28 °C [file MPP-22-117-s005.docx]

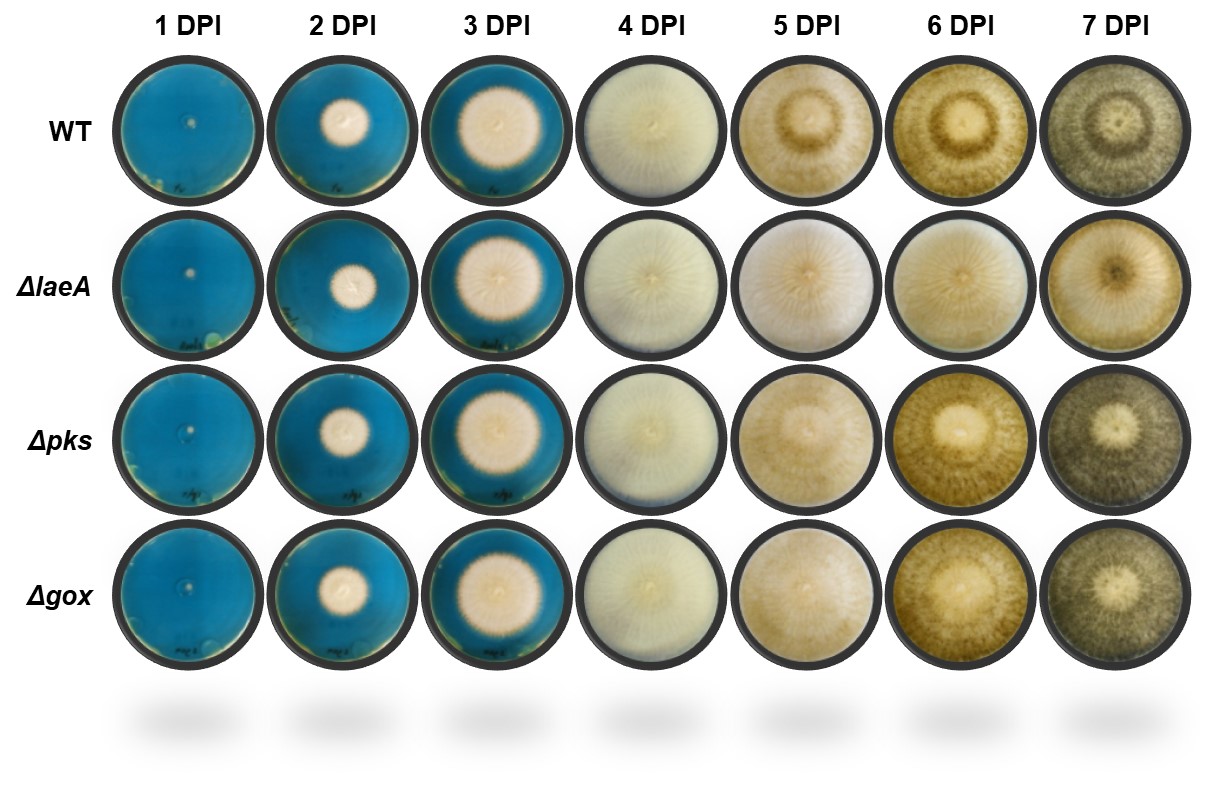


**Figure S5.** Growth phenotype of the WT, Δ*laeA, Δgox* and *Δpks* mutant strains of *A. carbonarius* in YES media under pH 4 at 28°C.
