# Supplementary material for: Functional roles of LaeA, polyketide synthase, and glucose oxidase in the regulation of ochratoxin A biosynthesis and virulence in Aspergillus carbonarius
Source: Mol Plant Pathol. 2020 Nov 10;22(1):117–29. doi: 10.1111/mpp.13013 (PMC7749749; doi:10.1111/mpp.13013)
Supplement: Supplementary file 6 — FIGURE S6 Physiological analyses of the wild type (WT) and ∆laeA strains of Aspergillus carbonarius. (a) Radial growth of the WT and ∆laeA strains on solid YES medium at 28 °C at pH 4. (b) Conidiation of the WT and ∆laeA strains on solid YES medium at pH 4. (c) Germination rates in the WT and ∆laeA strains were assessed in static YES broth at 28 °C at pH 4. Error bars represent the standard error of the mean (SEM) across three independent replicates. Asterisks denote significant differences between strains at p < .05 (Student’s t test) [file MPP-22-117-s006.docx]

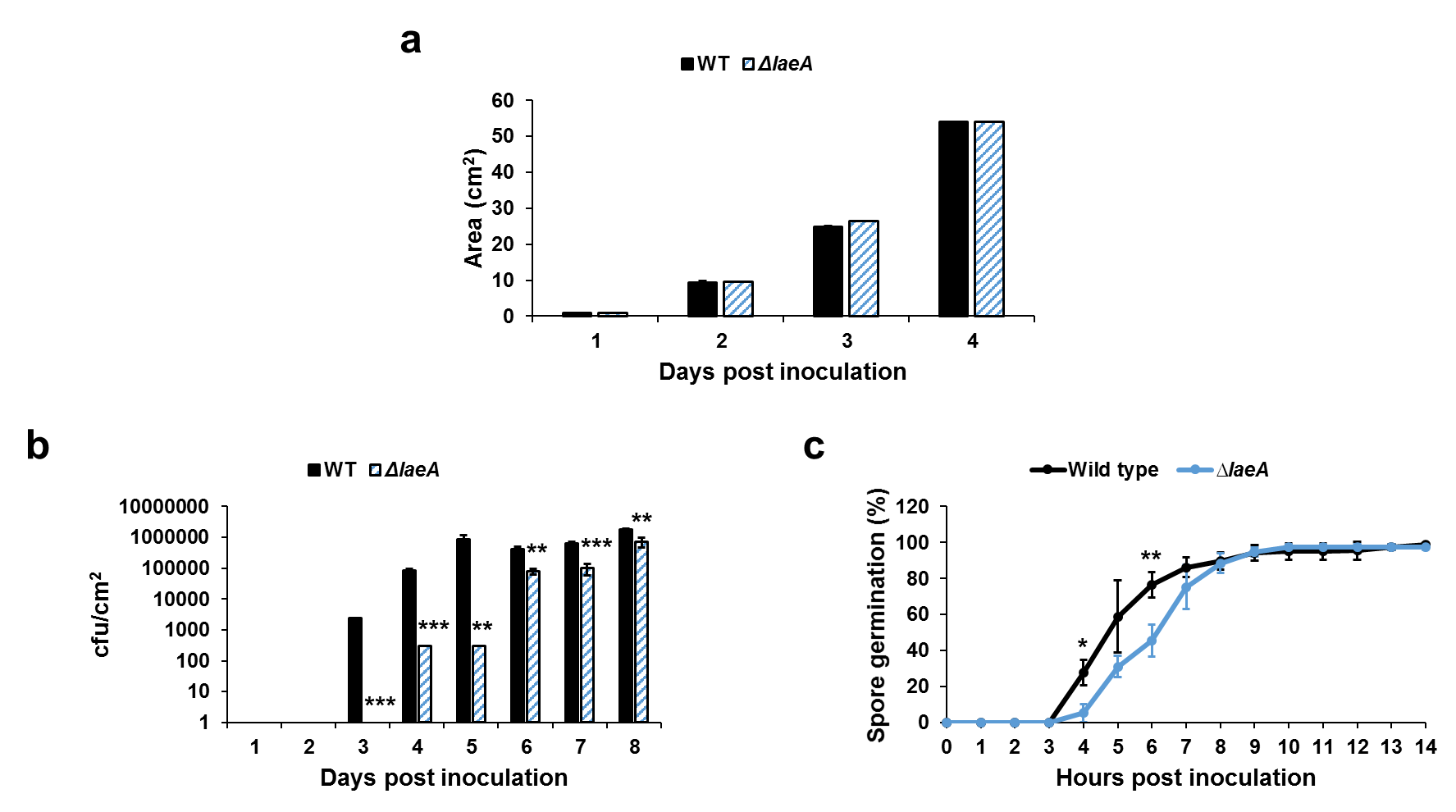


**Figure S6. Physiological analyses of the WT and *∆laeA* strains of *A. carbonarius*.** **(a)** radial growth of the WT and *∆laeA* strains on solid YES media at 28°C under pH 4.0; **(b)** conidiation of the WT and *∆laeA* strains on solid YES media at pH 4.0 **(c)** germination rates in the WT and *∆laeA* strains were assessed in static YES broth media at 28°C under pH 4.0. Error bars represent the standard error of the mean (SEM) across three independent replicates. Asterisks denote significant differences between strains at *p*<0.05 (Student's *t* test).
