# Supplementary material for: Functional roles of LaeA, polyketide synthase, and glucose oxidase in the regulation of ochratoxin A biosynthesis and virulence in Aspergillus carbonarius
Source: Mol Plant Pathol. 2020 Nov 10;22(1):117–29. doi: 10.1111/mpp.13013 (PMC7749749; doi:10.1111/mpp.13013)
Supplement: Supplementary file 7 — FIGURE S7 Effect of LaeA on OTA and GLA production in Aspergillus carbonarius: (a) OTA accumulation, (b) OTA cluster gene expression, (c) GLA accumulation, and (d) gox expression by the wild‐type and ∆laeA strains of A. carbonarius when grown on YES medium under pH 4 at 28 °C. Asterisks denote significant differences between strains (p < .05) [file MPP-22-117-s007.docx]

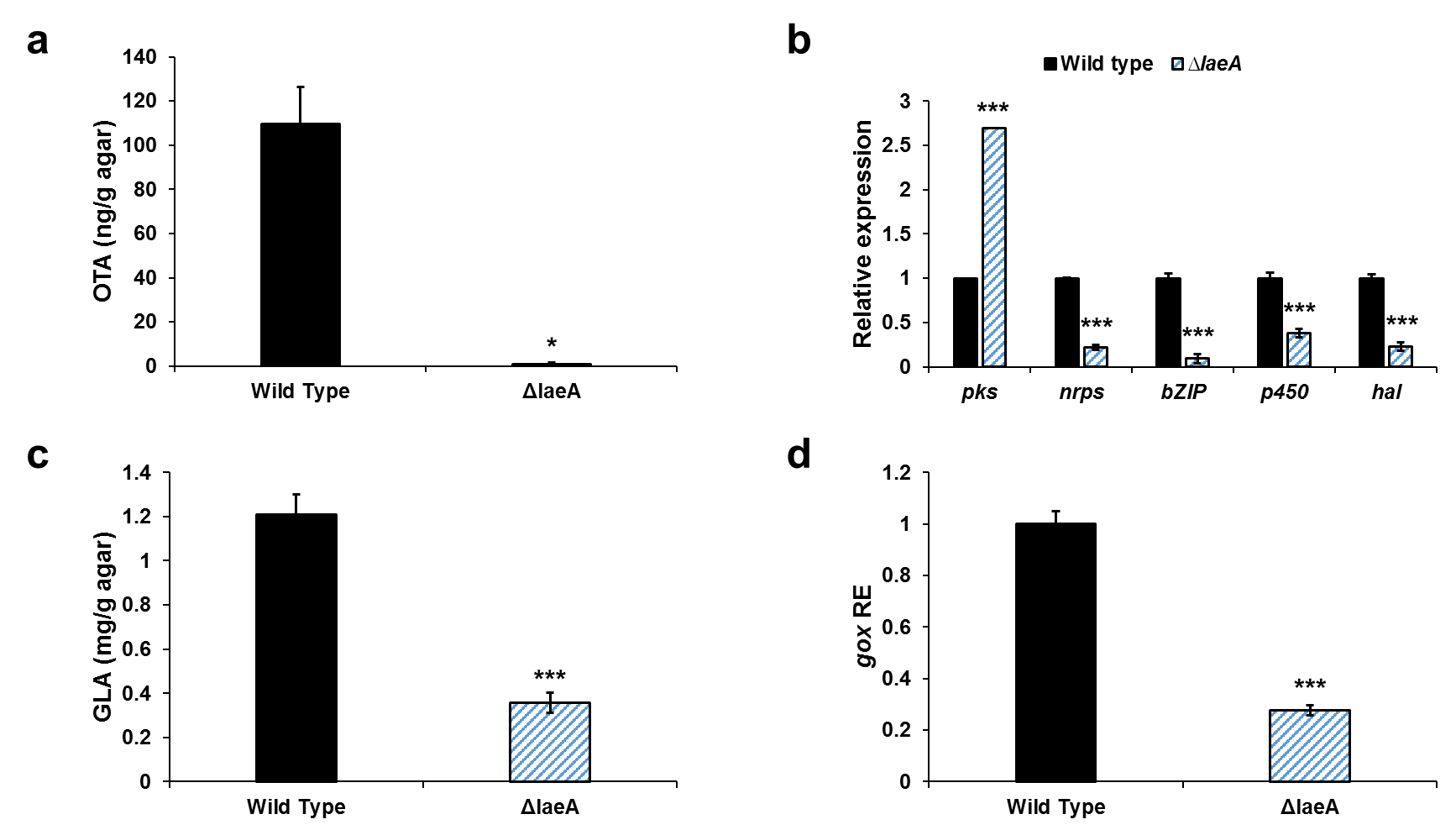


**Figure S7*.* Effect of LaeA on OTA and GLA production in *A. carbonarius.*** **(a)** OTA accumulation; **(b)** OTA cluster gene expression; **(c)** GLA accumulation, and **(d)** *gox* expression by the WT and ∆*laeA* strains of *A. carbonarius* when grown on YES medium under pH 4.0 at 28°C*.* Asterisks denote significant differences between strains (*p*<0.05).
