# Supplementary material for: Functional roles of LaeA, polyketide synthase, and glucose oxidase in the regulation of ochratoxin A biosynthesis and virulence in Aspergillus carbonarius
Source: Mol Plant Pathol. 2020 Nov 10;22(1):117–29. doi: 10.1111/mpp.13013 (PMC7749749; doi:10.1111/mpp.13013)
Supplement: Supplementary file 8 — FIGURE S8 Effect of PKS on Aspergillus carbonarius virulence and OTA synthesis in grapes: (a) OTA accumulation and (b, c) growth development of the wild type and ∆pks strains of A. carbonarius on freshly harvested grapes. (d) pH changes and (e) GLA accumulation in grape berries. Error bars represent the standard error of the mean (SEM) across three independent replicates. Different letters above the columns indicate statistically significant differences (p < .05) as determined using the Tukey’s honestly significant difference test [file MPP-22-117-s008.docx]

**Figure S8. Effect of PKS on *A. carbonarius* virulence and OTA synthesis in grapes.** **(a)** OTA accumulation and **(b, c)** growth development of the WT and *∆pks* strains of *A. carbonarius* on freshly harvested grapes. **(d)** pH changes, and **(e)** GLA accumulation in grape berries. Error bars represent the standard error of the mean (SEM) across three independent replicates. Different letters above the columns indicate statistically significant differences (*p*<0.05), as determined using the Tukey's honest significant difference test.
