# Supplementary material for: Functional roles of LaeA, polyketide synthase, and glucose oxidase in the regulation of ochratoxin A biosynthesis and virulence in Aspergillus carbonarius
Source: Mol Plant Pathol. 2020 Nov 10;22(1):117–29. doi: 10.1111/mpp.13013 (PMC7749749; doi:10.1111/mpp.13013)
Supplement: Supplementary file 9 — FIGURE S9 Effect of PKS on OTA and GLA production in Aspergillus carbonarius in vitro: (a) OTA accumulation, (b) laeA and OTA cluster gene expression, (c) GLA accumulation, and (d) gox gene expression by the wild type and ∆pks strains of A. carbonarius when grown in YES medium under pH 4 at 28 °C. Asterisks denote significant differences between strains (p < .05) [file MPP-22-117-s009.docx]

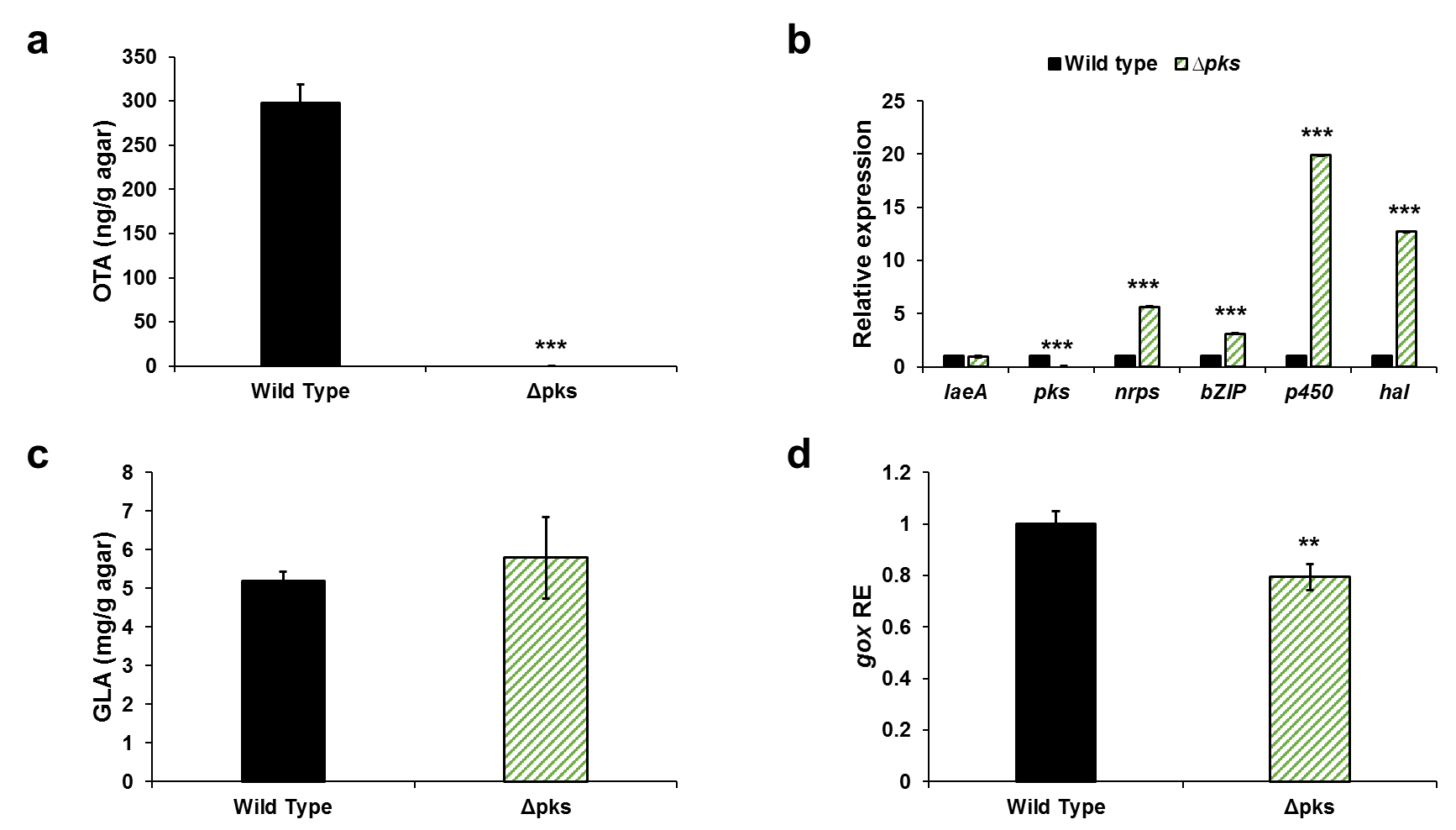


**Figure S9. Effect of PKS on OTA and GLA production in *A. carbonarius in vitro.*** **(a)** OTA accumulation, **(b)** *laeA* and OTA cluster gene expression, **(c)** GLA accumulation, and **(d)** *gox* gene expression by the WT and *∆pks* strains of *A. carbonarius* when grown in YES culture media under pH 4.0 at 28°C*.* Asterisks denote significant differences between strains (*p*<0.05).
