# Supplementary material for: Functional roles of LaeA, polyketide synthase, and glucose oxidase in the regulation of ochratoxin A biosynthesis and virulence in Aspergillus carbonarius
Source: Mol Plant Pathol. 2020 Nov 10;22(1):117–29. doi: 10.1111/mpp.13013 (PMC7749749; doi:10.1111/mpp.13013)
Supplement: Supplementary file 11 — FIGURE S11 Involvement of GOX in Aspergillus carbonarius virulence and OTA biosynthesis in grapes: (a) GLA production and (b, c) growth development of the wild type and ∆gox strains of A. carbonarius on freshly harvested grapes. (d) pH changes and (e) OTA accumulation in grape berries. Error bars represent the standard error of the mean (SEM) across three independent replicates. Different letters above the columns indicate statistically significant differences (p < .05) as determined using the Tukey’s honestly significant difference test. Asterisks denote significant differences between strains at p < .05 (Student’s t test) [file MPP-22-117-s011.docx]

**Figure S11. Involvement of GOX in *A. carbonarius* virulence and OTA biosynthesis in grapes.** **(a)** GLA production, and **(b, c)** growth development of the WT and *∆gox* strains of *A. carbonarius* on freshly harvested grapes. **(d)** pH changes, and **(e)** OTA accumulation in grape berries. Error bars represent the standard error of the mean (SEM) across three independent replicates. Different letters above the columns indicate statistically significant differences (*p*<0.05), as determined using the Tukey's honest significant difference test. Asterisks denote significant differences between strains at *p*<0.05 (Student's *t* test).
