# Supplementary material for: Functional roles of LaeA, polyketide synthase, and glucose oxidase in the regulation of ochratoxin A biosynthesis and virulence in Aspergillus carbonarius
Source: Mol Plant Pathol. 2020 Nov 10;22(1):117–29. doi: 10.1111/mpp.13013 (PMC7749749; doi:10.1111/mpp.13013)
Supplement: Supplementary file 12 — FIGURE S12 The effect of Acgox gene deletion on the production of organic acids by Aspergillus carbonarius: (a) GLA production and (b) citric acid accumulation by the WT and ∆gox strains of A. carbonarius grown on YES culture medium under pH 4.0 at 28 °C. Asterisks denote significant differences between strains at p < .05 (Student's t test) [file MPP-22-117-s012.docx]

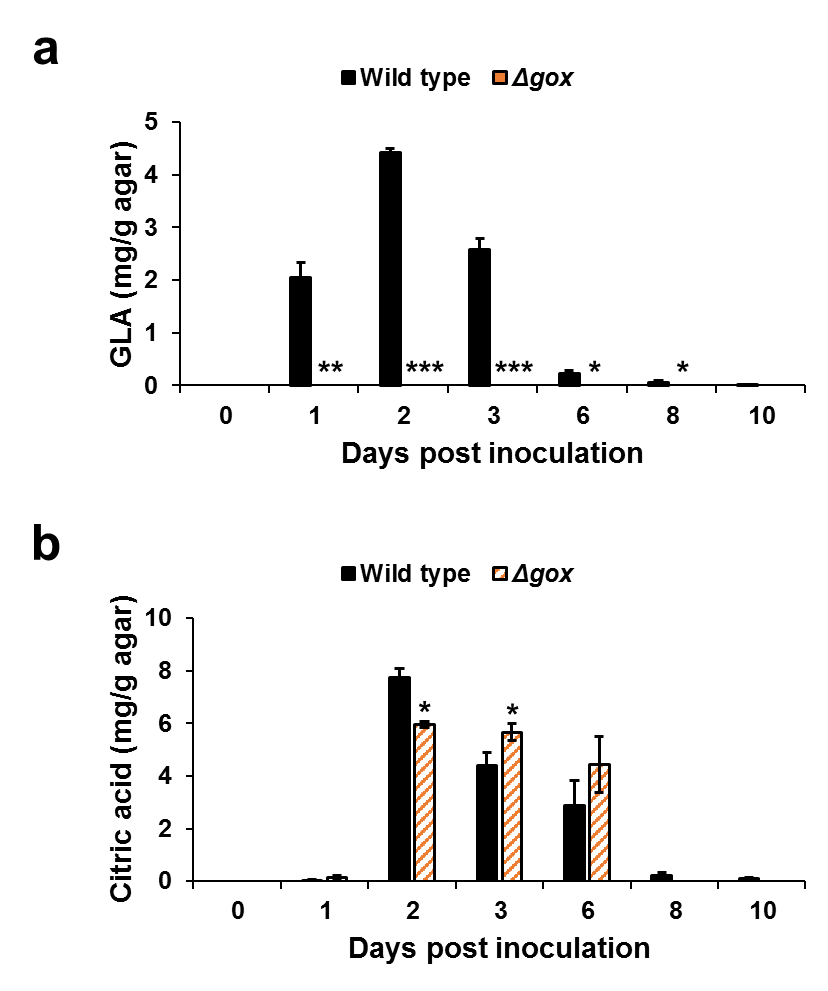


**Figure S12*.* The effect of *Acgox* gene deletion on the production of organic acids by *A. carbonarius*.** **(a)** GLA production, and **(b)** citric acid accumulation by the WT and *∆gox* strains of *A. carbonarius* grown on YES culture medium under pH 4.0 at 28°C*.* Asterisks denote significant differences between strains at *p*<0.05 (Student's *t* test).
