# Supplementary material for: Functional roles of LaeA, polyketide synthase, and glucose oxidase in the regulation of ochratoxin A biosynthesis and virulence in Aspergillus carbonarius
Source: Mol Plant Pathol. 2020 Nov 10;22(1):117–29. doi: 10.1111/mpp.13013 (PMC7749749; doi:10.1111/mpp.13013)
Supplement: Supplementary file 13 — TABLE S1 List of primers used in the study to create and confirm the mutant strains [file MPP-22-117-s013.docx]

**Table S1.** List of primers used in the study to create and confirm the mutant strains

| **#** | **Primer name** | **Sequence** | **Description** |
| --- | --- | --- | --- |
| 1 | LA | GGTCTTAAUCACCGGCCGAACCTTTGCTGC | Amplification of *AclaeA* promoter for construction of knockout vector |
| 2 | LB | GGCATTAAUCTCATTGCGGTCCCATTGCTCACTG |  |
| 3 | LC | GGACTTAAUCCGAAAGCATCGAGTCGTTCAGTC | Amplification of *AclaeA* Terminator for construction of knockout vector |
| 4 | LD | GGGTTTAAUCGGAGCCACGGCTCTCTGGTC |  |
| 5 | OTApks-F3 | GGGTTTAAUCGTGGTTGATGTGGTGAGTC | Amplification of *Acpks* promoter for construction of knockout vector |
| 6 | OTApks-R3 | GGACTTAAUCTATGTGGAAGGCAATGCAG |  |
| 7 | OTApks-F5 | GGCATTAAUGCTGTGCTGAACAGTTGACCC | Amplification of *Acpks* Terminator for construction of knockout vector |
| 8 | OTApks-R5 | GGTCTTAAUCGAATACATCGCCAACACAAAC |  |
| 9 | Gox up Fw | GGGTTTAAUTCTCCTTGTGCTGACCAACCG | Amplification of *Acgox* promoter for construction of knockout vector |
| 10 | Gox up Rv | GGACTTAAUGTTTACCAATCCCGCCGCGTC |  |
| 11 | Gox down Fw | GGCATTAAUAGGTGAGATGGAGTTGTTG | Amplification of *Acgox* Terminator for construction of knockout vector |
| 12 | Gox down Rv | GGTCTTAAUTTGGGATGGGTAGGGTATT |  |
| 13 | H-f1 | AGCTGCGCCGATGGTTTCTACAA | Amplification of hygromycin resistance gene internal sequence for verification |
| 14 | H-r1 | GCGCGTCTGCTGCTCCATACAA |  |
| 15 | L-f1 | CAATGGGACCGCAATGAGTC | Amplification of *AclaeA* internal sequence for verification |
| 16 | L-r1 | GGCTCGAAATCAATCTCCACC |  |
| 17 | P-f1 | CTAGACACGGCTCTGTTCGAAG | Amplification of *Acpks* internal sequence for verification |
| 18 | P-r1 | GGGATCCAGCTCAATGTTCAG |  |
| 19 | G-f1 | CACCGTCTTGGTCATCGAAAG | Amplification of *Acgox* internal sequence for verification |
| 20 | G-r1 | ATGTTCTCGACGGTGGTCATG |  |
| 21 | pacC f1 | TTTGAGCCCTCGTTGAACC | Amplification of *AcpacC* internal sequence for verification |
| 22 | pacC r1 | TGGTGTCCTGCATCTGCTG |  |
