# Supplementary material for: Functional roles of LaeA, polyketide synthase, and glucose oxidase in the regulation of ochratoxin A biosynthesis and virulence in Aspergillus carbonarius
Source: Mol Plant Pathol. 2020 Nov 10;22(1):117–29. doi: 10.1111/mpp.13013 (PMC7749749; doi:10.1111/mpp.13013)
Supplement: Supplementary file 14 — TABLE S2 Primer sequences used for quantitative reverse transcription PCR analysis [file MPP-22-117-s014.docx]

**Table S2.** Primer sequences used for qRT-PCR analysis

| **#** | **Primer name** | **Sequence** | **Description** |
| --- | --- | --- | --- |
| 1 | Btub_carF | GGACGAGATGGAGTTCACTGA | *β-tubulin* control gene |
| 2 | Btub_carR | CCTCTTGCTCAAGGACCTCCT |  |
| 3 | AcLaeA LI | CACCTATACAACCTCCGAACCAC | Velvet complex *laeA* gene |
| 4 | AcLaeA LJ | GGTTCGGCCAACCGACGACGCTG |  |
| 5 | Acgox f2 | TCGAACACTCTGGCATTGGA | Glucose oxidase (*gox*) gene |
| 6 | Acgox r2 | GTGGTTTGGTCCTGCAGGTT |  |
| 7 | PKS_carF | GGGATCGTACGATCTGGTGAT | OTA cluster polyketide synthase |
| 8 | PKS_carR | GGGAACACATGAGGTCAGGCT |  |
| 9 | NRPS_carF | CGGTAGAAAGACTGCAGTCCAT | OTA cluster non-ribosomal peptide synthetase |
| 10 | NRPS_carR | CGTCGGAATCCATTGCGCTGA |  |
| 11 | bZip_carF | CTCGACGGTTCGAGCCTTCT | OTA cluster bZip transcription factor |
| 12 | bZip_carR | GCATTCGCTCTAGCTGCTCGA |  |
| 13 | P450_carF | CCATCGTCTCCAGAGAATCAGT | OTA cluster cytochrome P450 monooxygenase |
| 14 | P450_carR2 | GGTCTCGTCGTGATGAATCAAG |  |
| 15 | HAL_carF | GCCAGTAGAGGGACAGCCAT | OTA cluster halogenase |
| 16 | HAL_carR | GCTGGAGGTGGTGGTTGAGA |  |
| 17 | AccbhA f1 | CTGTTGTCATTGACTCGAATTGG | Cellobiohydrolase A (*cbhA*) gene |
| 18 | AccbhA r1 | ATCCCAGGTGTTGCCAGTGT |  |
| 19 | AccbhB f1 | GAGCGACGACAGCAATTATGAG | Cellobiohydrolase B (*cbhB*) gene |
| 20 | AccbhB r1 | AAGGGAGGTTGGAGACATCCA |  |
| 21 | AceglB f1 | ATGGTAGATACAACGGCGAGATC | Endoglucanase B (*eglB*) gene |
| 22 | AceglB r1 | CCTGCAAGGTTCTCCCAGAA |  |
| 23 | AcxynB f1 | GTGGTCCGACGTGAGCAACT | Xylanase B (*xynB*) gene |
| 24 | AcxynB r1 | ATAGGTGATGTTCTGGGCACTTC |  |
| 25 | AcxlnD f1 | CGGTTCTCAGCCCTAGACATG | β-xylosidase D (*xlnD*) gene |
| 26 | AcxlnD r1 | CACGCTCTCCCCCGTGTA |  |
| 27 | AcpgaA f1 | CATCAACTCGGGTGAGAACATCT | Polygalacturonase A (*pgaA*) gene |
| 28 | AcpgaA r1 | TCGATGAAGGTAACGTTCTTGACA |  |
| 29 | AcpelA f1 | GTGGTGACGCTATCACTCTTGATG | Pectin lyase A (*pelA*) gene |
| 30 | AcpelA r1 | CCGATGCGAGCGGTAGTAAC |  |
| 31 | AcrhgA f1 | TTGGATGGGATTGATGTTTGG | Rhamnogalacturonase A (*rhgA*) gene |
| 32 | AcrhgA r1 | TGTTGTTGGCAGGACTCTTGAC |  |
